# Mouse precision-cut liver slices as an ex vivo model to study drug-induced cholestasis

**DOI:** 10.1007/s00204-022-03321-2

**Published:** 2022-06-16

**Authors:** R. E. H. Karsten, N. J. W. Krijnen, W. Maho, H. Permentier, E. Verpoorte, P. Olinga

**Affiliations:** 1 Pharmaceutical Analysis Research Group, Groningen Research Institute of Pharmacy, University of Groningen, Antonius Deusinglaan 1, 9713 AV Groningen, The Netherlands; 2 Analytical Biochemistry Research Group, Groningen Research Institute of Pharmacy, University of Groningen, A. Deusinglaan 16, 9713 AV Groningen, The Netherlands; 3 Pharmaceutical Technology and Biopharmacy Research Group, Department of Pharmaceutical Technology and Biopharmacy, Groningen Research Institute of Pharmacy, University of Groningen, Antonius Deusinglaan 1, 9713 AV Groningen, The Netherlands

**Keywords:** Bile acids, Chlorpromazine, Cyclosporin A, Glibenclamide, Bile acid transporters

## Abstract

Drugs are often withdrawn from the market due to the manifestation of drug-induced liver injury (DILI) in patients. Drug-induced cholestasis (DIC), defined as obstruction of hepatic bile flow due to medication, is one form of DILI. Because DILI is idiosyncratic, and the resulting cholestasis complex, there is no suitable in vitro model for early DIC detection during drug development. Our goal was to develop a mouse precision-cut liver slice (mPCLS) model to study DIC and to assess cholestasis development using conventional molecular biology and analytical chemistry methods. Cholestasis was induced in mPCLS through a 48-h-incubation with three drugs known to induce cholestasis in humans, namely chlorpromazine (15, 20, and 30 µM), cyclosporin A (1, 3, and 6 µM) or glibenclamide (25, 50, and 65 µM). A bile-acid mixture (16 µM) that is physiologically representative of the human bile-acid pool was added to the incubation medium with drug, and results were compared to incubations with no added bile acids. Treatment of PCLS with cholestatic drugs increased the intracellular bile-acid concentration of deoxycholic acid and modulated bile-transporter genes. Chlorpromazine led to the most pronounced cholestasis in 48 h, observed as increased toxicity; decreased protein and gene expression of the bile salt export pump; increased gene expression of multidrug resistance-associated protein 4; and accumulation of intracellular bile acids. Moreover, chlorpromazine-induced cholestasis exhibited some transition into fibrosis, evidenced by increased gene expression of collagen 1A1 and heatshock protein 47. In conclusion, we demonstrate that mPCLS can be used to study human DIC onset and progression in a 48 h period. We thus propose this model is suited for other similar studies of human DIC.

**Supplementary Information:**

The online version contains supplementary material available at 10.1007/s00204-022-03321-2.

## Introduction

Bile is the fluid produced by the liver to help digest lipids in the small intestine. Though strongly colored, bile in fact consists mostly of water (85%) supplemented by bile salts, bilirubin, fats and inorganic salts. A substantial amount of bile is produced every day by the human liver, on the order of 750–1000 mL (Pitt and Nakeeb 2017). Cholestasis, the inhibition of bile flow through intra- or extrahepatic bile ducts, can be caused by genetic and environmental factors (e.g. drugs), as well as pregnancy (Padda et al. 2011). The toxic effects of cholestasis in hepatocytes are most likely caused by the accumulation of substances in the liver tissue, bile ducts, or blood, which are normally excreted into the bile (e.g. bile salts, cholesterol, bilirubin and drug metabolites). This intra- and extrahepatic accumulation are thought to cause severe liver injury, as hepatocellular apoptosis and liver tissue necrosis. When this accumulation becomes chronic, cholestasis can lead to fibrosis and, ultimately, organ failure (Yang et al. 2013).

**Fig. 1:**
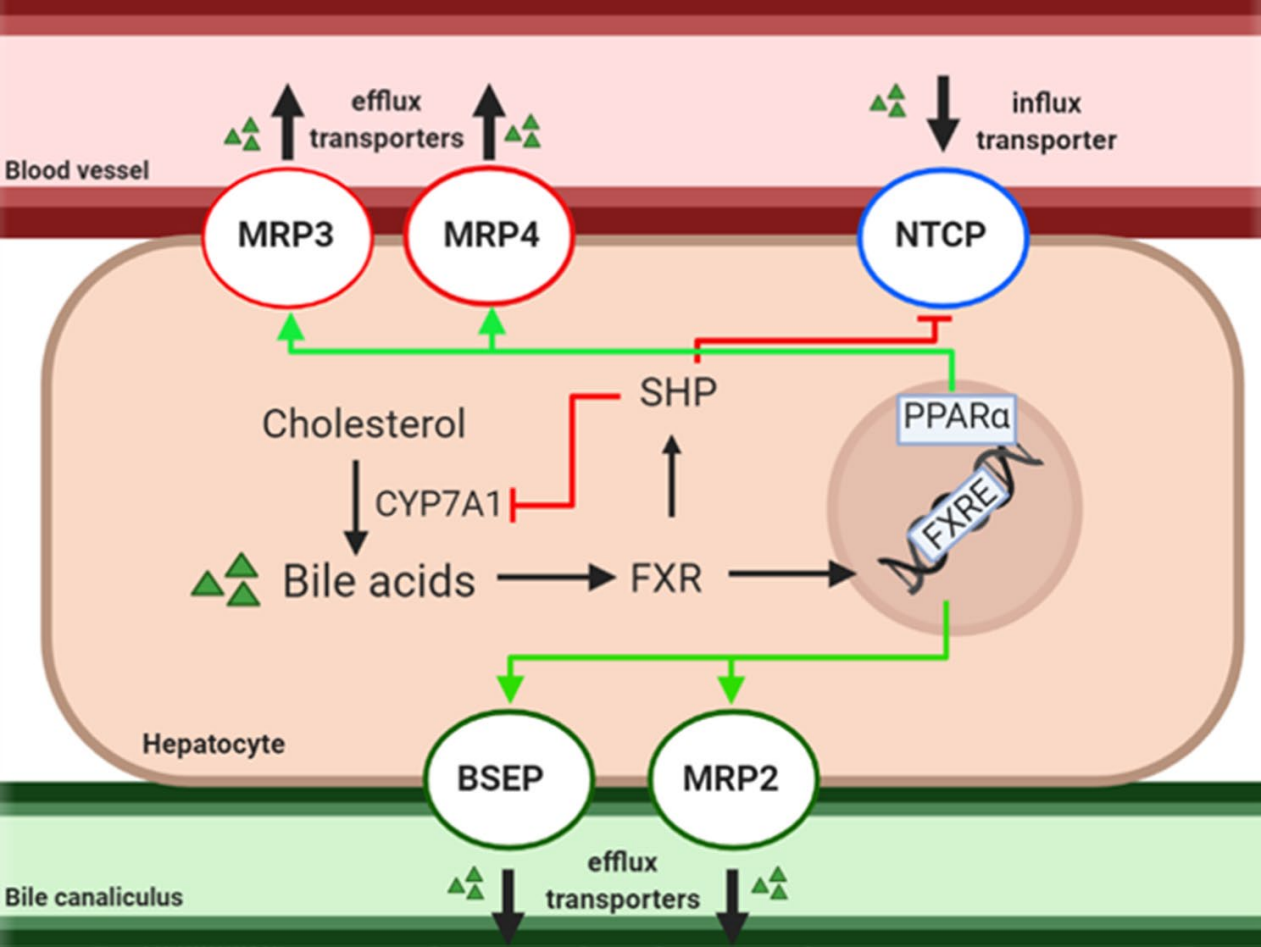
Simplified schematic representation of bile acid (BA) homeostasis in hepatocytes. Cholesterol is metabolized by the rate-limiting enzyme, cytochrome p450 7A1 (CYP7A1), to form BAs. These BAs can activate the nuclear farnesoid X receptor (FXR), which regulates BA homeostasis by activating FXR response elements (FXRE) on the DNA. This activation leads to increased BA transport via the canalicular efflux transporters, bile salt export pump (BSEP) and multidrug resistance-associated protein (MRP) 2. Moreover, FXR activates small heterodimer partner (SHP), which leads to the reduction of CYP7A1 and the influx transporter, sodium-taurocholate cotransporting polypeptide (NTCP). The expression of the basolateral efflux transporters, MRP3 and MRP4, is increased when intracellular BA levels rise, and is regulated by nuclear peroxisome proliferator-activated receptor α (PPARα), which in human is activated by FXRE (Pineda Torra et al. 2003; Claudel et al. 2005; Halilbasic et al. 2013)

In a healthy liver, maintenance of bile acid (BA) homeostasis involves transporters located on both the hepatic apical and basolateral membranes (Fig. 1). One of the two bile efflux transporters located on the apical membrane is the bile salt export pump (BSEP), which transports bile salts into the bile canaliculi to form bile. The other biliary transporter located on this membrane is the multidrug resistance-associated protein (MRP) 2. MRP2 transports drug metabolites and bile constituents, like bilirubin, into the bile canaliculi. The influx transporter, sodium-taurocholate co-transporting polypeptide (NTCP), which transports bile salts into the hepatocytes, is located on the basolateral membrane, as are the efflux transporters, MRP3 and MRP4, which transport bile salts and other solutes into the blood. (Köck and Brouwer 2012).

In a process known as drug-induced cholestasis (DIC), cholestatic drugs cause hepatocellular toxicity by the obstruction of bile flow in cells by inhibiting these biliary transporters, leading to perturbed BA homeostasis and accumulation of BAs in hepatocytes as a result (Fig. 1) (Cheng et al. 2016). Drugs can interfere either directly with bile transfer by inhibiting BSEP or other bile-salt transporters (Morgan et al. 2010, 2013; Zhang et al. 2016), or indirectly, by repressing transporter genes or the genes regulating these (Garzel et al. 2014; Vatakuti et al. 2017). Eventually, chronic cholestatic injury can progress into fibrosis, which is the development and deposition of extra fibrous connective tissue by e.g. activated hepatic stellate cells, which leads to undesirable remodeling of tissue architecture (Weiskirchen et al. 2018).

However, functional impairment of only one transporter will not result in cholestasis, as intrinsic defense mechanisms of hepatocytes are set in motion when cellular bile flow is disrupted (Fig. 1). These involve BA transport being taken over by non-impaired transporters through activation of nuclear receptor farnesoid X (FXR) and/or nuclear peroxisome proliferator-activated receptor α (PPARα) (Fig. 1) (Pineda Torra et al. 2003; Claudel et al. 2005; Köck and Brouwer 2012; Halilbasic et al. 2013). Thus, multiple bile transporters or bile homeostasis regulators need to be modulated for cholestasis to occur (Claudel et al. 2005; Köck and Brouwer 2012; Yang et al. 2013). A good model for the study of DIC should include hepatic defensive mechanisms by considering the expression and activity of all bile transporters and nuclear BA homeostasis regulators. Additionally, a good model should also include multiple cell types to show the progression of DIC; the activation of other cell types as a result of cholestatic injury (e.g. stellate cells).

Unfortunately, good predictive in vitro models for DIC, that consider all of the aforementioned proteins, are still lacking. Numerous studies have been performed using BSEP membrane vesicle assays to predict DIC in vitro (Morgan et al. 2010; Dawson et al. 2012). These studies reveal that not all cholestatic drugs inhibit BSEP, while some non-cholestatic drugs actually do. Therefore, BSEP inhibition appears insufficient as predictor of the cholestatic properties of a drug. Other studies using both MRPs and BSEP membrane vesicles provided a more accurate prediction of cholestatic properties (Morgan et al. 2013; Köck et al. 2014). Overall, these studies stressed the importance of BSEP and MRPs in the development of cholestasis, and thereby the multifactorial nature of cholestasis. We can conclude that a model that includes all cellular processes offers more accurate information for prediction of cholestasis than models focusing on the inhibition of individual transporters only.

Several studies investigating the mechanisms underlying DIC have been performed in more complex in vitro models, such as human hepatic cell cultures (Anthérieu et al. 2013), sandwich-cultured hepatocytes (Zhang et al. 2016) and spheroids (Hendriks et al. 2016; Shinozawa et al. 2021). Anthérieu et al. (2013) and Román et al. (2003) showed that not only direct inhibition of transporters but also internalization of BSEP at the canalicular membrane can result in cholestasis development. These findings indicate the need for a highly realistic tissue model that includes all cellular processes, multiple cell types and intact bile canaliculi.

The mouse precision-cut liver slices (PCLS) model may be an interesting model to capture the complexity of DIC. PCLS is in fact a miniature organ, providing an excellent rendition of the hepatic tissue. It is a multicellular system, maintaining cell heterogeneity, with an intact bile canaliculi structure (Elferink et al. 2004; Barth et al. 2006; de Graaf et al. 2007; Starokozhko et al. 2015). PCLS thus capture the complexity we need to study all processes and defense mechanisms in the cell that are affected by cell exposure to cholestatic drugs.

However, a challenge in this model is that the compositions of the mouse and human BA pools differ quite significantly. For both species, the primary BAs are cholic acid (CA) and chenodeoxycholic acid (CDCA) (Honda et al. 2020). These primary BAs are synthesized in the liver by the enzyme, cytochrome P450 7A1 (CYP7A1) (Claudel et al. 2005). Rodents, however, have an extra enzyme, CYP2C70, which synthesizes the hydrophilic primary BA, muricholic acid (MCA), from CDCA (Boer et al. 2019; Honda et al. 2020; Guo and Chiang 2020). In the liver, newly synthesized and recirculated BAs are conjugated with taurine or glycine to create more hydrophilic BAs. Humans conjugate BAs 60% to glycine and 35% to taurine (García-Cañaveras et al. 2012), whereas rodents almost exclusively conjugate BAs to taurine (97%) (Alnouti et al. 2008). In mice, taurine conjugation of the primary BA, MCA, creates a more hydrophilic and non-toxic BA pool than exists in human, which could explain why cholestatic toxicity is often overlooked in rodent models. There is an in vivo study (Boer et al. 2019) with knock-out mice lacking the rodent-specific enzyme CYP2C70 to create a more human-like BA pool; this model was established to improve translation of preclinical data to the human situation. However, this model still lacks glycine-conjugated BAs, since mice almost exclusively conjugate BAs to taurine. In this study, we aim to achieve a human-like mouse PCLS model to study DIC, by changing the BA pool in mouse PCLS through addition of a human-like BA mixture to the incubation medium. Our model can be used as a substitute for the knock-out mouse model (Boer et al. 2019), ultimately helping to refine and reduce animal testing.

We aim to provide a usable cholestasis disease model which allows us to monitor the onset and progression of DIC over shorter periods (up to 48 h). We have investigated multiple cellular processes and responses involved in the onset and progression of cholestasis, including cellular defense systems. Whereas other models tend to look at one aspect of DIC development at a time, we take a more comprehensive approach that takes advantage of a 3D biological model. In so doing, we describe the use of different techniques to elucidate several of the factors involved in the onset and development of cholestasis.

## Materials and methods

### Chemicals

Chlorpromazine hydrochloride (CPZ), Cyclosporin A (CSA), Glibenclamide (GB), and BA standards in sodium-salt or hydrated form, including CA, CDCA, DCA, LCA, UDCA, HDCA, TCA, TCDCA, THDCA, TDCA and GCA were all purchased from Sigma-Aldrich (the Netherlands) (See Table 1 for the full names of these BAs). Stock solutions were prepared by dissolving the BA in question in dimethyl sulfoxide (DMSO) (Sigma-Aldrich). Deuterium-labeled cholic acid (CA-D4) was bought from Toronto Research Chemicals (Toronto, Canada), for use as an internal standard in liquid chromatography–tandem mass spectrometry (LC–MS/MS) studies of intracellular BAs. Ethylene glycol-bis(2-aminoethylether)-*NNN*′*N*′-tetraacetic acid (EGTA), formic acid and D-glucose monohydrate were supplied by Sigma-Aldrich. Acetonitrile (HPLC grade) was acquired from Biosolve (the Netherlands). Purified water was prepared using a MilliQ Advantage A10 System (Millipore Corporation, the Netherlands). University of Wisconsin (UW) organ preservation solution was bought from DuPont Critical Care (Illinois, USA), Williams Medium E (1X, glutaMAX-1) and gentamicin (50 μg/ml) from Gibco (UK), and Hanks Balanced Salt Solution (HBSS) from Life Technology (California, USA).

**Table 1:** Composition of human-like BA mixture

Bile acids	Code	Medium concentration (µM)
Cholic acid	CA	9.12 (57.0%)
Chenodeoxy cholic acid	CDCA	0.31 (1.9%)
Deoxycholic acid	DCA	2.87 (17.9%)
Hyodeoxycholic acid	HDCA	0.43 (2.7%)
Lithocholic acid	LCA	0.16 (1.0%)
Ursodeoxycholic acid	UDCA	0.54 (3.4%)
Taurocholic acid	TCA	1.91 (12.0%)
Taurochenodeoxycholic acid	TCDCA	0.09 (0.6%)
Taurohyodeoxycholic acid	THDCA	0.14 (0.9%)
Sodium taurodeoxycholate hydrate	TDCA	0.40 (2.5%)
Glycocholic acid	GCA	0.03 (0.2%)
Total		16.0 (100%)

### Animals

Male C57BL/6 J mice, 8–11 weeks old, were provided by the Central Animal Facility of the University Medical Center Groningen. Mice were kept in a temperature- and humidity-controlled room in a 12-h light/dark cycle, with food and water ad libitum.

The experiments were approved by the Animal Ethical Committee of the University of Groningen (CCD number AVD105002017884) and were performed in accordance with EU Directive 2010/63/EU for animal experiments.

### Preparation of mouse PCLS

PCLS were prepared according to protocols described by de Graaf et al. (2010) and Karsten et al. (2019). In short, surgical procedures for liver excision were carried out under isoflurane/O_2_ anesthesia. Hereafter, the excised mouse liver was placed in ice-cold UW. Cores 5 mm in diameter were prepared from the liver with a biopsy punch, and 250-μm-thick PCLS were prepared with a Krumdieck Tissue Slicer (Alabama R&D, USA) filled with ice-cold saturated (95% O_2_, 5% CO_2_) Krebs Henseleit Buffer 1X (pH = 7.4). During the slicing process, liver cores and PCLS were stored in ice-cold UW.

### Incubation of mouse PCLS

PCLS were incubated as described previously for rat and human PCLS (de Graaf et al. 2010; Vatakuti et al. 2017; Starokozhko et al. 2017a; Karsten et al. 2019) with some modifications for mouse PCLS. In short, freshly prepared PCLS were transferred to 12-well plates (Greiner bio-one GmbH, Austria), one slice per well. Each well was filled with 1.3 mL pre-heated (37 ℃) and oxygenated (80%) Williams Medium E (1X, glutaMAX-1), which had been supplemented with 25 mM D-glucose monohydrate and 50 μg/ml Gentamicin (denoted as WME GG). Well plates were placed on a shake table in an incubator, and PCLS were incubated (37 ℃, 80% O_2_, 5% CO_2_, 15% N_2_, shaking 90 times/min) for 48 h in WME GG. PCLS were incubated under different conditions: (1) in the presence or absence of a human-like BA mixture (total BA concentration: 16 μM) (Table 1), in combination with (2) either CPZ (15, 20, 30 μM), CSA (1, 3, 6 μM), GB (25, 50, 65 μM) or vehicle DMSO. The DMSO concentration used was below 0.5% in the incubation medium, equivalent to DMSO concentrations in solutions prepared by dilution of BA stock solutions with WME GG. This led to the definition of four generally different incubation conditions, namely (a) no BAs or drug present (control) (b) only BAs present (BA control) (c) only drug present (at one of several possible concentrations) (d) both drug (at one of several possible concentrations) and BAs present. The composition of the human-like BA mixture was based on serum concentrations in mice and humans, measured by García-Cañaveras et al. (2012). The total concentration of the BA mixture was established based on a threshold concentration defined by a maximum of 10% loss in slice viability observed during incubation (37 ℃, 80% O_2_, 5% CO_2_, 15% N_2_, shaking 90 times/min) in the mixture.

### RNA isolation and cDNA synthesis

Three PCLS incubated under a given condition were pooled in a 1.5-mL Rnase-free test tube, snap-frozen in liquid nitrogen, and stored at – 80 °C until analysis. RNA was isolated using a FavorPrep Tissue Total RNA Mini Kit (Favorgen, Vienna, Austria). Subsequently, the RNA yield was quantified using a NanoDrop One instrument (Thermo Fisher Scientific, Massachusetts, USA). Isolated RNA was reverse-transcribed to cDNA using a cDNA synthesis kit (Reverse Transcription System, Promega, Benelux B.V.). The cDNA samples were subsequently loaded in the thermal cycler with a program set to 22 °C for 10 min, 42 °C for 15 min, and 95 °C for 5 min.

### Quantitative reverse-transcription PCR (qRT-PCR)

qRT-PCR was used to determine the relative mRNA expression levels for the mouse target genes: *Bsep* (Abcb11), *Mrp2* (Abcc2), *Mrp3* (Abcc3), *Mrp4* (Abcc4), *Ntcp* (Slc10a1), *Fxr* (Nr1h4), *Cyp7a1* (Cyp7a1), *Collagen type 1a1* (Col1a1), *Hsp47* (Serpinh1). Primer sequences of the genes tested are given in Supplementary Table S1. QRT-PCR was performed using, the dye, FastStart Universal SYBR Green Master Mix (Roche Diagnostics Netherlands B.V., Almere, the Netherlands) with the ViiA™ 7 Real-Time PCR System (Applied Biosystems, Bleiswijk, the Netherlands). The first cycle constituted 10 min at 95 °C, followed by 40 cycles of 15 s at 95 °C, 30 s at 60 °C and 30 s at 72 °C, followed by 1 cycle dissociation stage (15 s: 95 °C, 60 °C and 95 °C). A minimum of four mouse livers was used for each experiment, using three PCLS per condition. Samples were measured in duplicates. The comparative cycle threshold (CT) method (Schmittgen and Livak 2008), with Ywhaz (Ywhaz) as a reference gene, was used to calculate the fold change resulting from (1) a 48 h-BA control (incubation with BAs, no drug) compared to a 48 h-control (incubation with no drug, no BAs) (2) a 48 h-drug treatment (no BAs) compared to 48 h-control, or (3) a 48 h-treatment by drug in BA mixture compared to a 48 h-BA control. Results are expressed as log2(fold change).

### Western blot

The protein expression of Bsep (160 kDa) and Ntcp (57 kDa) was determined by western blotting as described previously by Ruigrok et al. (2018), but with minor modifications. In brief, the protein was extracted from three slices per treatment, and protein samples were denatured (75 °C for 10 min). Extracted protein (20 μg) was separated using SDS-PAGE with 7.5% gels. Gels were blotted onto 0.2-μm polyvinylidene fluoride membranes using a Trans-Blot Turbo Transfer System (Bio-Rad, Munich, Germany), set to ramp up to 25 V in 15 min, at a constant current of 2.5 A. Membranes were then treated for 1 h with a protein blocking agent to prevent non-specific binding of antibodies, and incubated at 4 °C overnight with Bsep primary antibody (monoclonal antibody rabbit anti-Bsep, 1:2500, Tebu-Bio, Le Perray-en-Yvelines, France), Ntcp primary antibody (polyclonal antibody, rabbit anti-Ntcp, 1:2000, Tebu-Bio), and vinculin (116 kDa) primary antibody (monoclonal antibody, mouse anti-Vinculin, 1:500, Santa Cruz, California, USA). The following day, membranes were incubated with matching secondary antibodies for 1 h (Goat anti-rabbit HRP (Bsep and Ntcp), 1:2000; Rabbit anti-mouse HRP (vinculin), 1:5000, Dako, Santa Clara). Proteins were then visualized by adding Clarity Western ECL blotting substrate (Bio-Rad, Munich, Germany) and using a ChemiDoc Touch Imaging System (BioRad, Munich, Germany) for imaging of the resulting chemiluminescence. Afterward, Bsep and Ntcp expression was normalized by vinculin expression.

### Quantification of intracellular BAs by LC–MS/MS

#### Standard and calibration preparation

A stock solution of each BA was prepared individually at a concentration of 1 mM in DMSO. A standard mixture of 11 BAs was prepared in acetonitrile: 40 µL of each BA was mixed, which gives a volume of 440 µL, and 3560 µL acetonitrile was added to get a concentration of 10 µM of each BA. Internal standard CA-D4 stock solution was prepared at a concentration of 2 mM in acetonitrile/water (1:1). The CA-D4 working solution was prepared at a concentration of 400 nM in acetonitrile/water (1:1.) A set of calibration solutions (500, 250, 100, 50, 25, 10, 5 and 1 nM) was prepared, all having a final internal-standard concentration of 200 nM in acetonitrile /water 1:1.

#### Instrumentation

A Shimadzu Nexera X2 HPLC system with binary pumps was interfaced to a Thermo Scientific TSQ Quantum ultra-triple quadrupole mass spectrometer. The BA analysis was performed by liquid chromatography-tandem mass spectrometry (LC–MS/MS). Xcalibur software version 2.0.7 by Thermo Scientific was applied in the data processing.

#### HPLC–MS analysis

A Kinetex XB-C18 (50 × 2.1 mm with 2.6 µm 100 Å particles; Phenomenex, Utrecht, the Netherlands) reversed-phase column was used to separate the BAs. The column and autosampler temperatures were set at 55 °C and 4 °C, respectively. The injection volume was 15 µL, and the flow was set at 0.3 mL/min. Mobile phase A consisted of water with 0.1% formic acid, mobile phase B consisted of acetonitrile with 0.1% formic acid. A linear gradient was used to separate the analytes: 0 min 3% B, 4 min 40% B, 7.0 min 60% B, 8 min 97% B, 10 min 97% B, 10.1 min 3% B, 14 min 3% B. mass spectrometric electrospray ionization (MS ESI) source parameters and multiple reaction monitoring (MRM) parameters were optimized with the standard mixture of BAs (4 × dilutes, final concentrations of 2.5 µM for each BA in acetonitrile/water 1:1), via direct infusion in negative ESI mode. The capillary voltage was 2.5 kV; vaporizer temperature, 400 °C; sheath gas pressure, 40; auxiliary gas pressure, 45; capillary temperature, 300 °C; and source temperature, 200 °C. The optimized parameters for each compound are listed in Table 2.

**Table 2:** Optimized MRM parameters for each bile acid

Compound	Parent mass	Fragment	Collision-induced dissociation (V)	Internal standard
	Q1	Q2		
*CA*	407.2	407.2	5	CA-D4
*LCA*	375.2	375.2	5	CA-D4
*UDCA*	391.2	391.2	5	CA-D4
*HDCA*	391.2	391.2	5	CA-D4
*CDCA*	391.2	391.2	5	CA-D4
*DCA*	391.2	391.2	5	CA-D4
*GCA*	464.4	74.0	40	CA-D4
*TCA*	514.3	80/124	50	CA-D4
*THDCA*	498.3	80/124	50	CA-D4
*TCDCA*	498.3	80/124	50	CA-D4
*TDCA*	498.3	80/124	50	CA-D4
*CA-D4*	411.2	411.2	5	

#### Sample extraction method

Intracellular BA concentrations in PCLS were measured by LC–MS/MS. Sample preparation for these measurements of intracellular BAs was performed as follows. After 47 h of incubation, PCLS were incubated (4 ℃, 95% O_2_, 5% CO_2_, shaking 90 times/min) in Ca^2+^-free and Mg^2+^-free Hanks Balanced Salt Solution (HBSS) supplemented with 5 mM EGTA for another hour to empty the bile canaliculi; this washing step is previously described by Starokozhko et al. (2017a). Hereafter, three slices for each condition were pooled in a 1.5-mL test tube, snap-frozen in liquid nitrogen and stored at -80 °C until analysis. Intracellular BAs from each set of three slices were extracted in 450 µL of acetonitrile + CA-D4 (440 µl acetonitrile, 10 µL CA-D4 working solution (2 µM)) using a Minibead-beater (Merck Life Science N.V., Amsterdam, the Netherlands) (five cycles of Minibead-beating (45 s) followed by cooling on ice (10 min)). Samples were subsequently centrifuged for 15 min at 15 °C and 13,000 rpm. The supernatant was collected in a new tube, and the samples were concentrated using a Concentrator plus centrifuge (Eppendorf, Germany). The sample pellet containing BAs was then reconstituted in 100 μL of acetonitrile:H_2_O (1:1) with a final internal standard concentration of 200 nM, and stored at -20 °C until analysis. The concentrations of CA, CDCA, DCA, LCA, UDCA, HDCA, TCA, TCDCA, THDCA, TDCA and GCA were determined with MRM-based LC–MS as described above.

### Viability assessment by ATP and protein content of PCLS

ATP is a well-established marker for PCLS viability and correlates to the number of viable hepatocytes scored with morphology (Westra et al. 2016). Protocols for measuring ATP and protein content in PCLS were developed and described previously (de Graaf et al. 2010; Karsten et al. 2019). In brief, after incubation, PCLS were individually snap-frozen in 1 mL of sonication solution (70% ethanol and 2 mM ethylenediaminetetraacetic acid (EDTA), pH = 10.9) using liquid nitrogen, and stored at -80 ℃ until analysis. ATP was extracted by homogenization, followed by the centrifugation of the homogenate (16,000*g* at 4 °C for 5 min). The supernatant was collected, and the pellet was left to dry. An ATP Bioluminescence Assay Kit (Roche Diagnostics, Mannheim, Germany) was used to determine ATP content in the supernatant. The dried pellet was used to determine the total protein content of the PCLS using the Bio-Rad DC Protein Assay (Bio-Rad, Munich, Germany). The obtained ATP values were then normalized for the total protein content of the slice. Based on luminescence measurements, the PCLS viability was calculated by comparing the luminescence obtained for (1) a 48 h-treatment by drug (no BAs) compared to 48 h-control, or (2) a 48 h-treatment by drug in BA mixture compared to 48 h-BA control (PCLS exhibited luminescence which was assumed equivalent to maximum viability for that mouse liver).

### Statistical analysis

A minimum of three mouse livers was used for each experiment, using PCLS in triplicate from each liver. GraphPad Prism 8.4.3 was used to perform statistical analysis, using a Student’s unpaired two-tailed *T*-test to compare two means, or a one-way ANOVA to compare multiple means. One-way ANOVA was followed by either Tukey’s multiple comparisons test to compare all means within a dataset, or Dunnett’s multiple comparisons test to compare all means with their own control mean. Nonlinear regression analysis was followed by an extra sum of squares *F*-test to compare two fits. mRNA expression levels are shown as Log2(fold induction) (− ΔΔCt), though the data was statistically analyzed using the cycle threshold (ΔCt) values. Differences between groups were considered significant when *p* < 0.05.

## Results

### Assessment of the mouse PCLS model to study drug-induced cholestasis (DIC)

In the first part of this study, mouse PCLS were incubated for 48 h in the presence or absence of the BA mixture, and then characterized to assess the mouse PCLS model to study human DIC. PCLS characterization involved measuring the expression of genes related to bile transport and BA homeostasis, the protein expression of bile uptake and export transporters, and the presence of intracellular BAs.

#### Gene expression

The expression of genes known to be involved in cholestasis development was measured with qRT-PCR (Table 3). These genes include the canalicular bile salt export transporters, *Bsep* and *Mrp2*, the basolateral (blood) bile salt export transporters, *Mrp3* and *Mrp4,* and basolateral uptake transporter, *Ntcp*. Gene expression data for the nuclear receptor regulating bile salt homeostasis, *Fxr,* and the main enzyme in the synthesis of primary BAs*, Cyp7a1*, were also included in this file. Finally, the gene expression of the early fibrosis markers, collagen type 1a1 (*Col1a1*), the main component of extracellular matrix, and heat shock protein 47 (*Hsp47*), which ensures proper collagen type 1a1 folding, were measured. These last two genes were of interest to ascertain if there is early onset of fibrosis evident in our cholestatic slices. As a result of cholestatic injury, among others, hepatocytes activate the stellate cells to increase the expression of *Col1a1* and *Hsp47*. Table 3 shows the log2(fold change) of gene expression for PCLS incubated for 48 h with and without BA addition, compared to freshly sliced PCLS incubated for 0 h. There was no significant difference in the expression of these 9 genes for slices incubated with or without added BAs for 48 h. Genes that were significantly downregulated compared to *t* = 0 (0 h-incubated PCLS) are highlighted in yellow. These include the genes for *Bsep*, *Mrp2*, *Ntcp*, *Fxr* and *Cyp7a1.* The gene expression of *Mrp3*, *Mrp4, Col1a1* and *Hsp47* was not significantly affected by incubation. The largest downregulation was observed for *Cyp7a1*, with a log2(fold change) of roughly -12, which means a factor of 144 decrease. The Ct-value of *Cyp7a1* after 48 h of incubation was above 30 cycles, or could not be determined. A large downregulation was observed as well for *Bsep* and *Ntcp*, with a log2(foldchange) of roughly -6 correlating to a factor of 36 decrease. *Mrp2* and *Fxr* were downregulated to a lesser extent, with a log2(fold change) of roughly -2, or a factor of 4 decrease.

**Table 3:**
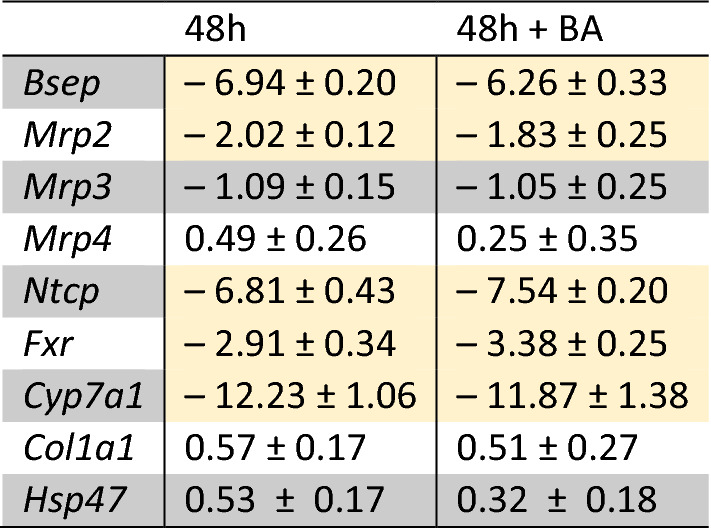
qRT-PCR of genes involved in bile acid (BA) homeostasis and cholestasis development, and their regulation after 48 h of incubation compared to 0 h-incubated PCLS

Values represent mean log2(fold change) ± SEM of 10 independent experiments (*n* = 10 mice, using three PCLS per mouse for each condition)

Significantly regulated genes with log2(fold change) below − 1.5 are highlighted in yellow

#### Protein expression

**Fig. 2:**
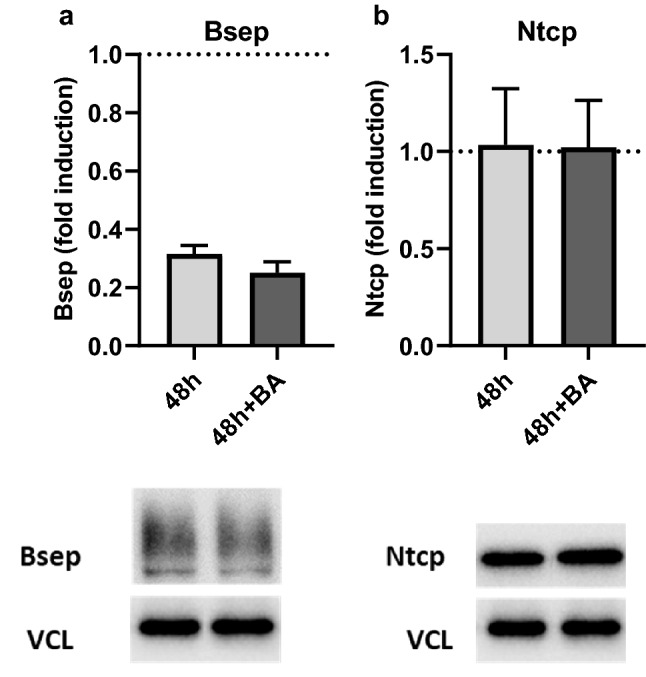
Western blot of **a** bile salt export pump (Bsep) and **b** sodium-taurocholate cotransporting polypeptide (Ntcp) at 48 h of incubation in the presence and absence of the humanized bile acid (BA) mixture. For each graph, a representative western blot band is shown. The mean band intensity of the protein was determined using vinculin (VCL) as a housekeeping protein, and the fold induction with respect to the 0 h-control (dotted line) was calculated. Data are shown as the mean of three independent experiments (*n* = 3 mice) ± SEM, using three slices per mouse for each condition. An unpaired two-tailed Student’s *T*-test was used to compare the 48 h-control to the 48 h-control with BAs. No statical analysis could be performed comparing 48 h-control to 0 h-control, because the 0 h-control contained the samples of the three mice pooled

Besides gene expression, we also investigated the protein expression of Bsep and Ntcp in our PCLS model, based on their large loss in gene expression over 48 h and their importance generally in cholestasis, whether drug-induced or not. Figure 2 shows the protein expression of Bsep (Fig. 2a) and Ntcp (Fig. 2b) per condition: 48 h-incubated slices and 48 h-incubated slices in medium containing the humanized BA mixture. Obtained protein expression values were corrected with the loading control vinculin and calculated relatively to the 0 h-control (indicated in the graph with a dotted line). Both transporters are still expressed after 48 h of incubation with or without the addition of BAs, although Bsep exhibits a significant drop after 48 h of incubation to a value approximately one-third that of the 0 h-incubated slices.

#### Concentrations of intracellular BAs

In this third PCLS characterization study, we quantitatively determined the intracellular concentrations of the 11 BAs of interest in our humanized BA mixture. However, it should be noted that the total amount of BAs may vary in the PCLS, especially when slices are fresh (0 h-incubation) and still contain physiological concentrations of BAs, such as the rodent-specific BA, MCA. MCA accounts for 40% of the total amount of BAs present in mice (Alnouti et al. 2008). Therefore, the total amount of BAs at 0 h is probably higher than we have measured, given that we have not comprehensively profiled all the BAs present.

**Fig. 3:**
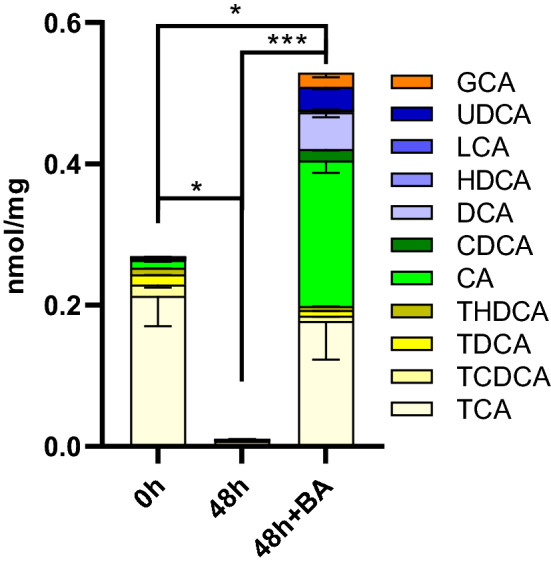
LC–MS/MS of bile acids (BAs) extracted from PCLS incubated for 0 h and 48 h in the presence or absence of the humanized BA mixture. The intracellular concentrations of 11 different BAs were determined (see Table 2). Primary BAs in green (CA and CDCA), secondary BAs in blue (UDCA, HDCA, DCA and LCA), taurine-conjugated BAs in yellow (TCA, TCDCA, TDCA and THDCA), and glycine-conjugated BA in orange (GCA). Data are presented as absolute values (nmol BA per mg protein) ± SEM. Graphs represent cumulative values of the mean values of individual BAs for five independent experiments (*n* = 5 mice) using three slices per mouse for each condition. One-way ANOVA was performed, followed by Tukey’s post hoc test to compare all means (*p < 0.05 ***p < 0.001)

The total BA content in PCLS incubated for 48 h diminished by 91–97% compared to a 0 h-incubated PCLS (Fig. 3, supplementary Table S2). The addition of the humanized BA mixture to the medium led to a compositional change of the intracellular BA content, and a twofold increase in measured intracellular BA compared to 0 h-incubated PCLS (Fig. 3). We measured a reduction of taurine-conjugated BAs (from 94 to 37.6%), an increase in glycine-conjugated BA (from 0.1 to 3.8%), and a large increase in unconjugated BAs (from 5.9 to 58.7%) (Fig. 3, supplementary Table S2).

### Implementing the mouse PCLS model to study drug-induced cholestasis (DIC)

For the following section, mouse PCLS were incubated for 48 h with or without the humanized BA mixture in the medium, and in the presence or absence of various concentrations of the well-known cholestatic drugs CPZ, CSA and GB. These experiments were performed to further elucidate the mechanistic events underlying DIC.

#### Viability

**Fig. 4:**
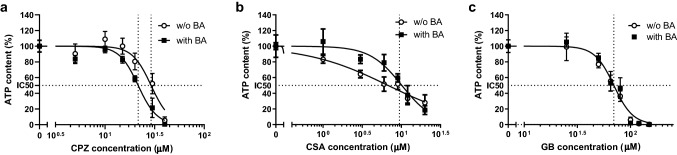
Normalized concentration–response curves of PCLS incubated for 48 h with either **a** chlorpromazine (CPZ), **b** cyclosporin A (CSA), or **c** glibenclamide (GB) in the presence (“with”) or absence (“w/o”) of the humanized bile acid (BA) mixture. Concentrations tested: CPZ: 5, 10, 15, 20, 30, 40 µM; CSA: 1, 3, 6, 9, 12, 20 µM; GB: 25, 50, 65, 80, 100, 120, 150 µM. Data are presented as the mean ± SEM of 3 independent experiments (n = 3 mice), using three slices per mouse for each condition. Nonlinear regression analysis is performed to find the best curve-fit and 50% inhibitory concentration (IC50). The IC50 of CPZ is 29 µM (w/o BA) and 22 µM (with BA); IC50 of CSA is 9.5 µM (with and w/o BA); and the IC50 of GB is 70 µM (with and w/o BA)

Using an ATP-luminescence assay, the viability of mouse PCLS was assessed after incubating the PCLS for 48 h, in the presence or absence of increasing concentrations of cholestatic drug, with or without the BA mixture (Fig. 4). All three cholestatic drugs showed a concentration-dependent decrease in ATP content. To establish the concentrations of each drug to be used to induce cholestasis in our PCLS model, we used a nonlinear regression analysis of the best-curve fit to calculate the inhibitory concentrations corresponding to losses in the viability of 5% (IC5), 25% (IC25), and 50% (IC50) for each cholestatic compound with and without BAs (Fig. 4, supplementary Table S3). The drug concentrations chosen based on this data were 15, 20 and 30 μM (CPZ, Fig. 4a); 1, 3 and 6 μM (CSA, Fig. 4b); and 25, 50 and 65 μM (GB, Fig. 4c). The three concentrations correspond to non-toxic (less than 5% viability loss), low toxicity (25% viability loss), and moderate toxicity (50% viability loss).

Furthermore, we tested if the addition of the BA mixture to the medium enhanced drug toxicity. Nonlinear regression analysis followed by an extra sum of squares F-test showed that the BA mixture enhanced the toxicity of CPZ considerably, with significantly (*p* = 0.0007) lower IC5, IC25 and IC50 in the presence of BAs (Fig. 4a, supplementary Table S3). This trend was not observed for CSA or GB (Fig. 4b, c).

At concentrations below the IC50 in the absence of added BAs, CSA treatment of PCLS resulted in a shallow curve with a significantly (p = 0.006) different Hill slope compared to with BAs, having a value between -1 and 0 (Fig. 4b, supplementary Table S3). A significantly lower IC10 (*p* = 0.002) and IC25 (*p* = 0.001) (Fig. 4b, supplementary Table S3) were observed compared to CSA treatment with the BA mixture.

#### Gene expression

**Fig. 5:**
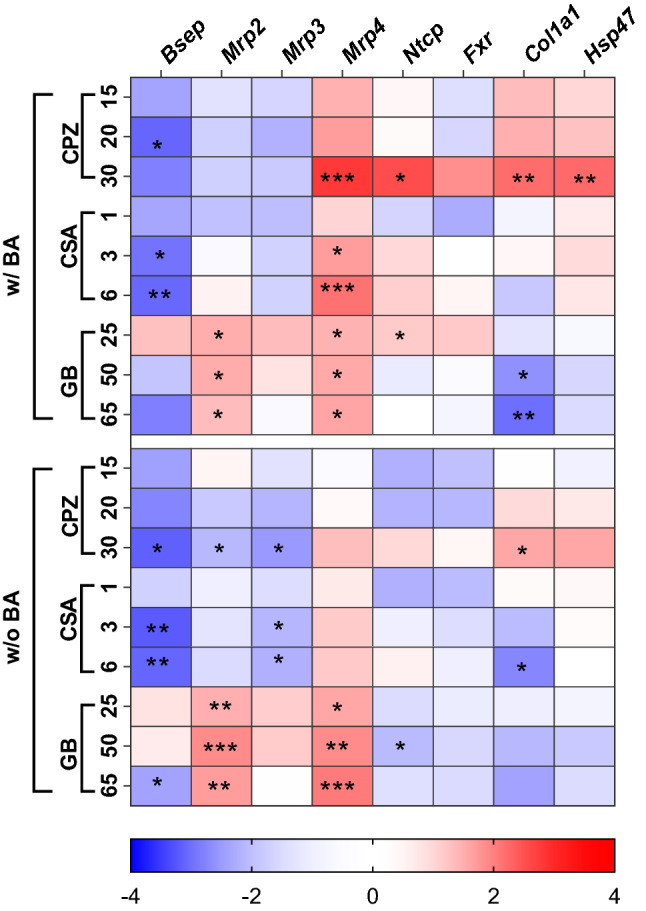
Heatmap of qRT-PCR results for the expression in PCLS of genes involved in DIC development, at non- low- and moderately-toxic concentrations of the cholestatic drugs, chlorpromazine (CPZ), cyclosporin A (CSA), or glibenclamide (GB), in the presence (w/) or absence (w/o) of the humanized bile acid (BA) mixture after 48 h of incubation. Blue shades indicate downregulation, while red shades indicate upregulation, compared to appropriate controls. The data are presented as log2(fold change) of 4 independent experiments (*n* = 4 mice), using three slices per mouse for each condition. Statistical analysis was performed as one-way ANOVA with Dunnett’s post hoc test, with * indicating a *p* < 0.05; **p < 0.01; ***p < 0.001

We measured the expression of genes involved in DIC development after 48 h of incubation in the presence or absence of the BA mixture, and with or without a non-, low- or moderately toxic concentration of drug. The results are presented in a heatmap (Fig. 5). Differentially regulated genes are colored red when upregulated and blue when downregulated, compared to an appropriate PCLS incubation control with no drug, and with or without BAs. Statistical analysis comparing drug treatments at the same drug concentration, with and without BA addition, did not reveal any significant differences in gene expression as a function of the presence or absence of BAs. However, drug treatment with or without the addition of BAs compared to an appropriate PCLS incubation control with no drug, and with or without BAs, did lead to significant differences. These differences are indicated as p-values in Fig. 5. CPZ reduces *Bsep*, *Mrp2* and *Mrp3* gene expression in the presence and absence of BAs, not significantly for *Mrp2* and *Mrp3* in the presence of BAs*.* CSA reduces *Bsep* expression in a concentration-dependent manner in the presence and absence of a BA mixture, and a small but significant decrease in *Mrp3* expression is observed in the absence of BAs. GB increases *Bsep* expression at 25 µM and decreases *Bsep* expression significantly at 65 μM when BAs are present. In contrast to CPZ and CSA, GB increases the expression of *Mrp2*, more abundantly in the absence of BAs. All three drugs increase *Mrp4* expression in a concentration-dependent manner. When treated with CPZ and CSA, this increase in *Mrp4* is more pronounced in the presence of BAs; when treated with GB, this increase is more pronounced in the absence of BAs. The highest concentration of CPZ and the lowest concentration of GB increase the expression of *Ntcp* significantly when BAs are present. A slight increase in *Ntcp* expression could also be observed for CSA when BAs are present. On the contrary, GB appears to decrease the *Ntcp* expression in the absence of BAs.

The early fibrosis markers, *Col1a1* and *Hsp47*, were also investigated in this study. In the presence of BAs, the highest concentration of CPZ significantly increased *Col1a1* and *Hsp47* expression. These changes were less pronounced in the absence of BAs, with only *Col1a1* showing a significant increase in expression. Treatment with CSA and GB, on the other hand, reduced *Col1a1* expression in a concentration-dependent manner, both with and without BAs present. Following incubation with 50 μM and 65 μM GB, there was a significant decrease in *Col1a1* expression in the presence of BAs; this *Col1a1* expression was also decreased in the absence of BAs, though this decrease was not statistically significant. In the absence of BAs, the highest concentration of CSA significantly decreased *Col1a1*.

#### Protein expression

**Fig. 6:**
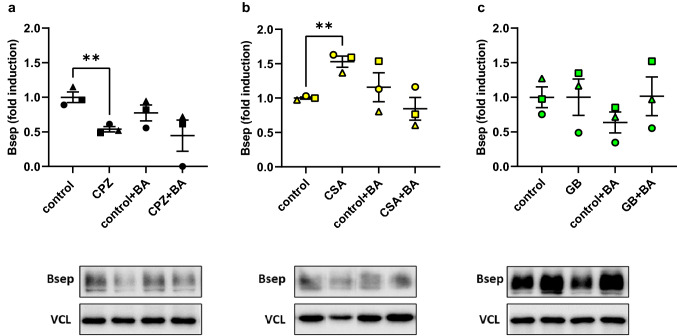
Western blot of bile salt export pump (Bsep) for mouse PCLS incubated for 48 h with a non-toxic concentration of either **a** chlorpromazine (15 µM) (CPZ), **b** cyclosporin A (1 µM) (CSA), or **c** glibenclamide (25 µM) (GB) in the presence or absence of the humanized bile acid (BA) mixture. For each graph, a representative western blot band is shown (complete bands are shown in supplementary Fig. S1–3). The mean band intensity of the protein was determined using vinculin (VCL) as a housekeeping protein, and the fold induction with respect to the 48 h-control was calculated. Data are shown as mean of three independent experiments (*n* = 3 mice; round, square, and triangle) ± SEM, using three slices per mouse for each condition. An unpaired two-tailed Student’s *T*-test was used to compare any given treatment with its own control. (*p < 0.05 and **p < 0.01)

**Fig. 7:**
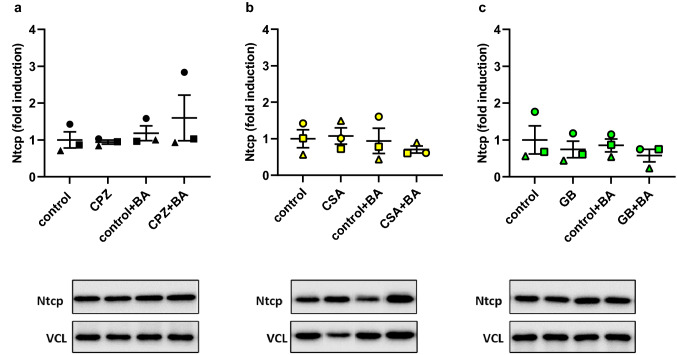
Western blot of sodium-taurocholate cotransporting polypeptide (Ntcp) in mouse PCLS incubated for 48 h with a non-toxic concentration of either **a** chlorpromazine (15 µM) (CPZ), **b** cyclosporin A (1 µM) (CSA), or **c** glibenclamide (25 µM) (GB) in the presence or absence of the humanized bile acid (BA) mixture. For each graph, a representative western blot band is shown (complete bands are presented in supplementary Fig. S1–3). The mean band intensity of the protein was determined using vinculin (VCL) as a housekeeping protein, and the fold induction with respect to the 48 h-control was calculated. Data are shown as the mean of three independent experiments (*n* = 3 mice; round, square, and triangle) ± SEM, using three slices per mouse for each condition. An unpaired two-tailed Student’s *T*-test was used to compare any given treatment with its own control (*p < 0.05 and **p < 0.01)

We investigated the changes in protein expression of Bsep (Fig. 6) and Ntcp (Fig. 7) after 48 h of incubation with a non-toxic concentration of cholestatic drug and in the presence or absence of the BA mixture. Semi-quantitative western blot analyses of Bsep protein expression showed a significant decrease in band intensity caused by a non-toxic concentration (15 μM) of CPZ (Fig. 6a) compared to the control with no drug and no BAs. This decrease was less pronounced in the presence of the BA mixture, in which a small decrease was as well observed with only the BA mixture compared to the control with no BAs. Treatment of PCLS with a non-toxic concentrations of CSA (1 μM) (Fig. 6b) increased the band intensity of Bsep significantly compared to the control with no drug and no BAs. There were no significant changes observed in the presence of BAs. Non-toxic concentrations of GB (25 μM) (Fig. 6c) did not induce any significant changes in Bsep protein expression levels. The protein expression of Ntcp remained quite stable after incubation with the three individual drugs (Fig. 7).

#### Concentrations of intracellular BAs

**Fig. 8:**
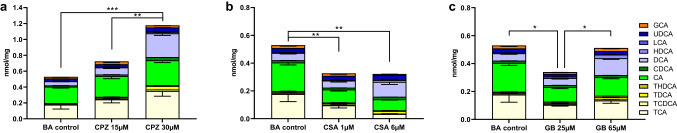
LC–MS/MS data for the intracellular concentrations of 11 different BAs in PCLS incubated for 48 h in the presence of the humanized bile acid (BA) mixture and with or without a cholestatic drug: **a** chlorpromazine (CPZ) (15, 30 µM), **b** cyclosporin A (CSA) (1, 6 µM), or **c** glibenclamide (GB) (25,65 µM). Primary BAs are shown in green (CA and CDCA), secondary BAs are shown in blue (UDCA, HDCA, DCA and LCA), taurine-conjugated BAs are shown in yellow (TCA, TCDCA, TDCA and THDCA), and the glycine-conjugated BA is shown in orange (GCA). Data are presented as absolute values (nmol BA per mg protein) ± SEM; individual values can be found in Table 4. Graphs present cumulative values of mean values of individual BAs for 5 independent experiments (*n* = 5 mice), using three slices per mouse for each condition. One-way ANOVA was performed, followed by Tukey’s post hoc test to compare all means (*p < 0.05; **p < 0.01; ***p < 0.001)

We measured intracellular BAs with LC–MS/MS after 48 h of incubation in the presence or absence of the humanized BA mixture, and with or without a non- or moderately toxic concentration of drug. Incubation of PCLS with CPZ led to an intracellular accumulation of BAs (Fig. 8a, Table 4). Moreover, all the concentrations of individual BAs increased as well (Fig. 8a, Table 4). A significant increase was observed for TDCA, UDCA, HDCA, CA, CDCA and DCA at the highest CPZ concentration (30 μM) (Fig. 8a, Table 4). Treatment with CSA (Fig. 8b) and GB (Fig. 8c) reduced or did not affect the total intracellular BA concentrations compared to the control. The main effect was a reduction in the intracellular concentrations of TCA and CA (Table 4). However, not all BA concentrations were decreased or unchanged; for example, a significant increase was observed for TDCA, HDCA and DCA following incubation with the highest concentration of CSA (6 μM) and GB (65 μM) (Fig. 8b, c, Table 4).

**Table 4:** Intracellular concentrations of 11 different bile acids (BAs) in PCLS incubated for 48 h in the presence of the humanized BA mixture, and with or without a cholestatic drug: chlorpromazine (CPZ) (30 µM), cyclosporin A (CSA) (6 µM), or glibenclamide (GB) (65 µM). BAs are listed from low to high hydrophobicity

pmol/mg protein	BA control	CPZ 30 μM	CSA 6 μM	GB 65 μM
THDCA	5.5 ± 0.4 (1.0%)	9.0 ± 3.0 (0.8%)	4.4 ± 1.0 (1.4%)	11.4 ± 2.6 (2.2%)
TCA	177.7 ± 54.8 (33.6%)	351.2 ± 67.0 (29.8%)	33.3 ± 2.0 (10.3%)*	135.2 ± 19.9 (26.4%)
TCDCA	7.2 ± 1.2 (1.4%)	18.2 ± 5.3 (1.5%)	3.6 ± 0.4 (1.1%)*	8.6 ± 1.2 (1.7%)
TDCA	8.2 ± 1.3 (1.6%)	44.3 ± 5.1 (3.8%)****	18.8 ± 2.3 (5.9%)**	14.6 ± 0.9 (2.9%)**
GCA	20.3 ± 6.8 (3.8%)	26.1 ± 3.5 (2.2%)	5.7 ± 1.0 (1.8%)	22.4 ± 6.1 (4.4%)
UDCA	31.9 ± 3.2 (6.0%)	46.3 ± 3.0 (3.9%)*	28.9 ± 1.6 (9.0%)	24.5 ± 3.9 (4.8%)
HDCA	2.5 ± 1.7 (0.5%)	13.6 ± 3.9 (1.2%)*	12.9 ± 1.8 (4.0%)**	25.7 ± 1.5 (5.0%)****
CA	206.2 ± 17.6 (39.0%)	314.1 ± 29.7 (26.7%)*	78.8 ± 4.9 (24.5%)****	132.3 ± 11.3 (25.9%)**
CDCA	16.3 ± 1.8 (3.1%)	41.3 ± 6.4 (3.55%)**	19.3 ± 2.6 (6.05%)	14.8 ± 1.3 (2.9%)
DCA	52.0 ± 7.0 (9.8%)	297.9 ± 25.1 (25.3%)****	106.9 ± 18.8 (33.2%)*	121.3 ± 8.6 (23.7%)****
LCA	1.5 ± 0.3 (0.3%)	14.9 ± 8.6 (1.3%)	8.9 ± 4.3 (2.8%)	0.9 ± 0.3 (0.2%)
Total	529.3 ± 82.3 (100%)	1177.0 ± 113.2 (100%)	321.7 ± 29.7 (100%)	511.7 ± 43.6 (100%)

Data for individual intracellular BA concentrations are expressed as mean ± SEM of five independent experiments (*n* = 5 mice), with the percentage of the total amount of BA in parentheses

A one-way ANOVA was performed, followed by a Dunnett’s post hoc test to compare data for different drug concentrations with their control (*p < 0.05; **p < 0.01; ***p < 0.001, ****p < 0.0001) (Data for additional drug concentrations can be found in Supplementary Table S4)

**Fig. 9:**
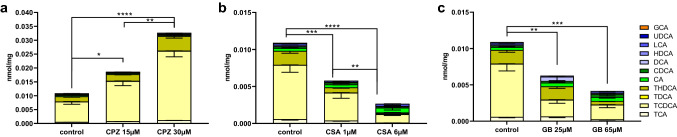
LC–MS/MS data for the intracellular concentration of 11 different BAs in PCLS incubated for 48 h in the absence of the humanized bile acid (BA) mixture, and with or without a cholestatic drug: **a** chlorpromazine (CPZ) (15, 30 µM), **b** cyclosporin A (CSA) (1, 6 µM), or **c** glibenclamide (GB) (25, 65 µM). Primary BAs are shown in green (CA and CDCA), secondary BAs are shown in blue (UDCA, HDCA, DCA and LCA), taurine-conjugated BAs are shown in yellow (TCA, TCDCA, TDCA and THDCA), and the glycine-conjugated BA is shown in orange (GCA). Data are presented as absolute values (nmol BA per mg protein) ± SEM; individual values can be found in Supplementary Table S5. Graphs present cumulative values of mean values of individual BAs for five independent experiments (*n* = 5 mice), using three slices per mouse for each condition. One-way ANOVA was performed followed by Tukey’s post hoc test to compare all means (*p < 0.05; **p < 0.01; ***p < 0.001)

Figure 9 and supplementary Table S5 show the LC–MS/MS data of PCLS treated with drugs in the absence of humanized BA mixture. Clearly, the BA concentrations presented in this figure are extremely low. The BA pool contains only BAs that originate from the mouse PCLS after 48 h of incubation. However, the PCLS incubated in the absence of the BA mixture still show the same trend as in the presence of the BA mixture. CPZ treatment (Fig. 9a) increased the total intracellular concentration of BAs, while treatment with CSA (Fig. 9b) and GB (Fig. 9c) led to a decrease in intracellular concentrations of BAs.

## Discussion

Several studies have made use of different biological models based on mice to study DIC, including mouse liver slices (Szalowska et al. 2013), an in vivo mouse model (Yu et al. 2017), and primary mouse hepatocytes (Van den Hof et al. 2017). Despite the fact that intracellular BA accumulation is a clear marker for cholestasis, none of these studies considered the composition of BA on DIC development. Some studies do take into account the important role of human BAs in DIC in vitro (Chatterjee et al. 2014; Hendriks et al. 2016; Ogimura et al. 2017; Van Brantegem et al. 2020; Shinozawa et al. 2021) and in vivo (Boer et al. 2019). Most in vitro models, however, do not include the tissue complexity of the PCLS model, which allows for the more faithful tissue response required in multifactorial conditions like cholestasis (Vatakuti et al. 2017; Starokozhko et al. 2017a). Moreover, the PCLS model has a higher throughput compared to the in vivo mouse model and is a very promising approach for reducing and refining animal experiments, due to the dramatically reduced amounts of mouse tissue required for individual experiments. In the PCLS model high levels of oxygen and glucose (25 mM glucose, 80% oxygen) are widely utilized, for both human and rodent tissue (de Graaf et al. 2007; Starokozhko et al. 2015, 2017b). These concentrations are not representative of in vivo conditions. Yet, studies have indicated that an excess of nutrients is required to maintain liver slices viable in culture for 48 h (Drobner et al. 2000; Vanhulle et al. 2001; Martin et al. 2002). High oxygen concentration is needed because the oxygen delivery to the tissue is not as efficient in vitro as it is in vivo. In vitro, oxygen is delivered without oxygen carriers and exclusively via diffusion. The 25 mM of glucose was shown to be required to maintain glycogen levels in rat PCLS (Vanhulle et al. 2001). As no insulin is added to the incubation medium, only part of the glucose added is likely taken up. While glucose uptake transporters in the liver are not sensitive to insulin, a large part of glucose uptake in the liver is still insulin-dependent (Suzuki et al. 1991; Kemas et al. 2021). Therefore, in this study we supplemented the media with 25 mM glucose and 80% oxygen to ensure optimal incubation conditions and to support comparability with earlier research (Vatakuti et al. 2017; Starokozhko et al. 2017a).

The primary goal of this study was to optimize the mouse PCLS model as an ex vivo model to better reflect the human liver ex vivo for the study of drug-related human toxicity. We have assessed the impact of incubating PCLS with a humanized BA mixture and several cholestatic drugs, to further increase our mechanistic understanding of DIC. Studies have been performed with this ex vivo PCLS cholestasis model with human (Vatakuti et al. 2017) and rat tissue (Starokozhko et al. 2017a), using physiological human BA and rat BA mixtures, respectively, for slice incubation, but not yet in mice. An advantage of the mouse PCLS model, is the possibility to modify the mouse DNA by the use knock-out mice, or small interference RNA (Ruigrok et al. 2018) to further elucidate mechanisms behind human DIC development. In this work, we developed a humanized mouse PCLS model by making use of a human-like BA mixture for slice incubation. This study additionally increases our fundamental understanding of several mechanisms underlying DIC, as discussed below.

### Assessment of the mouse PCLS model to study drug-induced cholestasis (DIC)

The objective of the first part of this study was to assess the mouse PCLS model to study human DIC. In DIC, drugs obstruct bile flow through inhibition of the ability of BA transporters to transfer BA into and out of the cell, leading to the perturbation of BA homeostasis through the intracellular accumulation of BA in hepatocytes, and consequently causing liver toxicity as hepatocellular apoptosis and necrosis. Intracellular BA accumulation is thus a primary characteristic of cholestasis, making the expression of functional BA transporters in a model for DIC an essential first criterion. A second essential criterion of a mouse model to study human DIC is an intracellular BA composition that is humanized, that is, consists of a BA mixture that is physiologically relevant for humans.

Concerning the first criterion that BA transporters be expressed, the loss of transporter expression during incubation is a frequently reported limitation of various in vitro models for cholestasis (Rippin et al. 2001; Borlak and Klutcka 2004; Soldatow et al. 2013), due mainly to cell dedifferentiation. To observe if this limitation also exists in our model, we measured gene expression of BA transporters in PCLS, as well as the associated gene regulators. Incubation of PCLS for 48 h with and without BAs resulted in a significant decrease of *Ntcp*, *Bsep*, *Mrp2* and *Fxr* expression, with the largest decrease observed for *Ntcp* and *Bsep* (Table 3*)*. Since *Ntcp* is the main BA uptake transporter and *Bsep* is the main BA export transporter, we also measured their respective protein expression by western blot to confirm that BAs can be transported in and out of the cells. Our results show that the protein expression of Ntcp is maintained during incubation, while protein expression of Bsep decreases (Fig. 2). The activity of the individual transporters after incubation was not investigated. However, the intracellular BA concentrations were investigated, including the concentrations of TCDCA and TCA. Since Bsep has a high affinity for TCDCA and TCA transport (Green et al. 2000; Kosters and Karpen 2008), these BAs are often used in functional activity assays of the mouse Bsep protein (Noe et al. 2001; Kis et al. 2009). LC–MS/MS data showed that these bile acids do not accumulate over a period of 48 h compared to the 0 h-incubated PCLS (Fig. 3, supplementary table S2). The concentrations of these BAs are similar or decreased compared to the 0 h-control, TCDCA decreases from 15.8 to 7.2 pmol/mg protein and TCA decreases from 213.0 to 177.7 pmol/mg protein. Indicating that the Bsep that is present is functional. Additionally, the Bsep protein is abundantly expressed in the 0 h-control sample and clear bands were observed for the Bsep protein as well after 48 h of incubation, showing that Bsep expression might be less but is still clearly present after 48 h of incubation. Decreased but functional Bsep might be beneficial to observe DIC in a 48 h timeframe.

Besides looking at genes related to BA homeostasis, we also studied the toxicity of DIC, which progresses into fibrosis (Hirschfield et al. 2010). To observe the effect of BA as such on the early onset of fibrosis, we examined the regulation of the early fibrosis markers *Col1a1* and *Hsp47*. Our gene expression data show that 48 h-incubation and the addition of BAs to the incubation medium did not change the regulation of these genes.

The second criterion for our model is the accumulation of intracellular human BAs during incubation to ensure a human-like BA pool for induction of DIC. This is because DIC is generally not observed in mouse models, due primarily to the more pronounced hydrophilicity of the mouse BA pool compared to the human pool (Boer et al. 2019). Our results (Fig. 3) show that the total BA content in PCLS incubated for 48 h diminished by 91–97%, compared to a 0 h-incubated PCLS. A similar decrease was observed by Starokozhko et al. (2017a) in rat PCLS. This large decrease is likely due to the unavailability of BAs that normally circulate in the bloodstream, and due to the downregulation of *Cyp7a1* expression, which translates to the enzyme Cyp7a1 and is essential for the synthesis of primary BAs. Since there is no de novo BA synthesis and no access to a bloodstream with circulating BAs in our model, we incubated PCLS with specific BAs to create a more human-like BA pool. The addition of a human-like BA mixture to the incubation medium resulted in a marked compositional change and a twofold increase of intracellular BAs, compared to PCLS analyzed directly after slicing. After 48 h of incubation with a humanized BA mixture, we observed reduced taurine-conjugated BAs and increased the glycine-conjugated BA and secondary BAs. We conclude that during incubation, the mouse PCLS BA pool better reflects the human BA pool that is required to study DIC development.

### Implementing the mouse PCLS model to study drug-induced cholestasis (DIC)

**Fig. 10:**
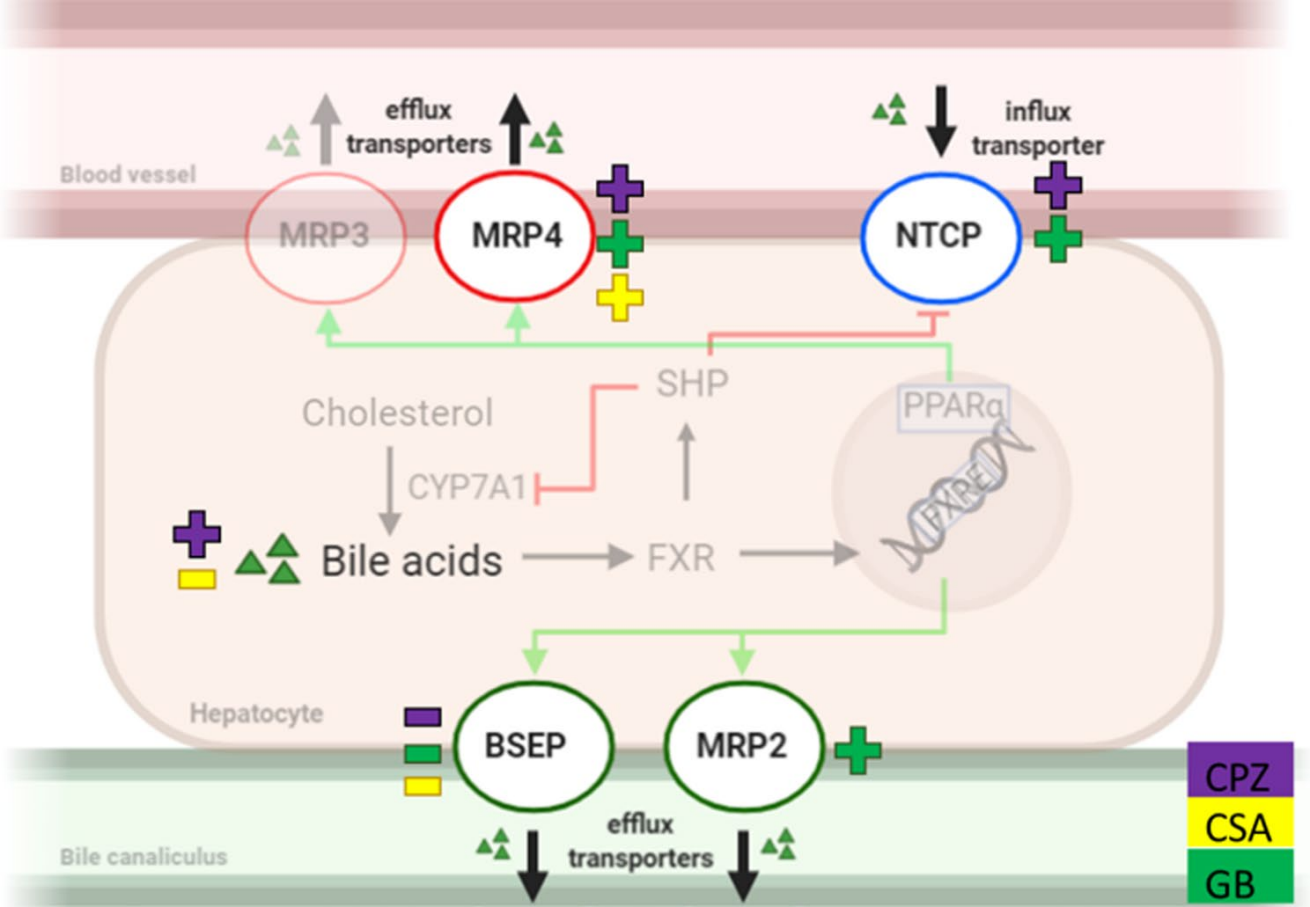
Simplified schematic summary of qRT-PCR and LC–MS/MS results after slice incubation with chlorpromazine (CPZ, purple), cyclosporin A (CSA, yellow), glibenclamide (GB, green) in the presence of the humanized bile acid (BA) mixture. Increased gene expression or increased intracellular concentration BAs (+), decreased gene expression or decreased intracellular concentration BAs (−). Multidrug canalicular efflux transporters bile salt export pump (BSEP), and multidrug resistance-associated protein (MRP), sodium-taurocholate cotransporting polypeptide (NTCP)

After confirming that all transporters are present and we successfully mimicked the human BA pool in mouse PCLS, we implemented the mouse PCLS model to study DIC. This section aimed to develop a cholestatic model by using a drug known to induce cholestasis in humans. Slices were incubated with one of three model cholestatic drugs, CPZ, CSA or GB, in the presence or absence of the humanized BA mixture. A graphic summary of the main results can be found in Fig. 10.

#### PCLS viability after incubation with model cholestatic drugs

First, we assessed drug toxicity using viability data. Our results (Fig. 4a) showed enhanced toxicity when incubating PCLS with CPZ and a humanized BA mixture. In contrast, incubation of PCLS with a humanized BA mixture and either CSA or GB did not result in enhanced toxicity when compared to the incubations without the human BA mixture (Fig. 4b, c). In rat PCLS enhanced toxicity was observed for CPZ and GB only in the presence of BAs, reaching severe toxicity with 36 µM CPZ and 180 µM GB (Starokozhko et al. 2017a). In mouse PCLS, GB was toxic at a considerably lower concentration, with and without BA, compared to rat PCLS. In primary human hepatocytes, 50% toxicity was observed with 102.6 µM of GB in the presence of a BA mixture (Ogimura et al. 2017), we observed this already at 70 µM of GB. These data suggest interspecies differences between mouse, human, and rat toxicity for GB, this is also shown in vivo (Pfizer 2020). The study with human hepatocytes was not conducted without the addition of BAs, thus if the toxicity observed with mice is comparable to human toxicity still needs to be investigated in human PCLS. The IC50 value found with CPZ treatment (22–29 µM CPZ) is comparable to the IC50 value found in human PCLS (Vatakuti et al. 2017), in which a similar decrease of 40% in ATP content was found with 30 µM CPZ. Also, this IC50 value corresponds with the IC50 value found in human hepatocytes, which was between 3 and 35 µM CPZ (Parmentier et al. 2013). The IC50 value found with CSA treatment was 9.5 µM CSA. Similar results were found in a study with human hepatocytes which showed that a concentration above 10 µM CSA with BAs was needed to observe DIC (Chatterjee et al. 2014). The concentration found in human PCLS was higher (Vatakuti et al. 2017), in this study, a 45% decrease in ATP content was found with 15 µM CSA. These drug concentrations in human PCLS were only tested without the BA mixture present, therefore a true comparison could not be made and still needs to be investigated in human PCLS.

When PCLS are treated with CSA, BAs appear to affect the dose–response curve of CSA (Fig. 4b, supplementary Table S3). In the presence of BAs, the dose–response curve has an expected Hill slope value less than − 1. In the absence of BA, however, the dose–response curve of CSA has a Hill slope having a value larger than − 1 (absolute value less than 1), which is significantly different. This difference may be related to the chemical properties of CSA. Indeed, CSA is a highly hydrophobic compound and solubilization by BAs is required for its absorption (Stojančević et al. 2013; Pharmacists Association 2021). Additionally, data in human hepatocytes showed that the toxicity of CSA is highly dependent on the presence of BAs (Parmentier et al. 2013; Sharanek et al. 2015). The study of Parmentier et al. (2018) showed similar differences in dose–response curves with and without the addition of BAs, supporting that BAs are necessary for the proper solubilization of CSA.

#### Gene expression

After assessing viability, we measured the gene expression of all the aforementioned proteins that play a role in the development of cholestasis, namely those responsible for intracellular BA uptake (influx transporters) and export (efflux transporters), as well as the BA homeostasis regulator, *Fxr* (Fig. 5). Individual incubation of PCLS with all three drugs resulted in downregulation of the canalicular bile exporter *Bsep* and upregulation of the basolateral bile exporter *Mrp4*. Decreased *Bsep* expression by cholestatic drugs is in concordance with previous studies in mouse, rat and human PCLS (Szalowska et al. 2013; Vatakuti et al. 2017; Starokozhko et al. 2017a). However, the observed decrease in *Bsep* expression due to incubation of mouse PCLS with GB was absent in rat PCLS under similar conditions (Starokozhko et al. 2017a). This further substantiates the interspecies variation between mice and rats.

In contrast to the direct effect of drug on *Bsep* gene expression, the increase in *Mrp4* gene expression could be related to an adaptive response of the cell (Wagner et al. 2003). An increase in the number of Mrp4 transporters is expected to result in an increased total concentration of BAs in the blood, which is a characteristic of DIC (Zollner et al. 2006; Halilbasic et al. 2013). *Mrp4* expression is increased via PPARα and constitutive androstane receptor (CAR) activation upon increased concentrations of intracellular BAs (Halilbasic et al. 2013). The large increase in *Mrp4* expression in the presence of BA and CPZ or CSA could therefore indicate a response to rising intracellular BAs and DIC development. However, with GB, the largest increase in *Mrp4* expression is observed in the absence of BAs, which indicates a direct effect of GB on *Mrp4* gene expression. The observed increase in *Mrp4* gene expression due to incubation with GB has not been reported before, to the best of our knowledge. All three compounds are known to inhibit MRP4 protein functionality in human membrane vesicles (Köck et al. 2014). Although we did not measure Mrp4 function, the increase in *Mrp4* gene expression could be the cell’s response to impairment of Mrp4 on a protein level. GB markedly increased the gene expression of the canalicular bile exporter Mrp2 as well, with the increase again most pronounced in the absence of BAs. As with the effect of GB on *Mrp4* expression, the induction of *Mrp2* gene expression by GB has not been reported in the literature, to the best of our knowledge. However, some studies have shown that cholestatic drugs inhibit or induce MRP2 on a protein level, where GB appears to stimulate MRP2 activity (Pedersen et al. 2008; Morgan et al. 2013). Since the increase in *Mrp2* gene expression is more pronounced in the absence of BAs, we speculate that the *Mrp2* upregulation is a response of the cell to GB, rather than to rising intracellular BAs.

No significant expressional changes were observed for *Fxr* in our work*,* which was unexpected since *Fxr* is the main regulator of BA homeostasis, and BAs serve as ligands that activate the Fxr protein, likely resulting in an upregulation of its gene expression (Claudel et al. 2005). Therefore, the absence of an effect on *Fxr* gene expression could be due to the low concentration of intracellular BAs measured with LC–MS/MS after CSA and GB treatment (Fig. 8b, c). In contrast, PCLS incubation with CPZ led to increased intracellular BA concentrations, with the largest increase in intracellular BAs due to incubation with 30 µM CPZ and the humanized BA mixture (Fig. 8a). qRT-PCR data (Fig. 5) somewhat supports the idea that increased intracellular BA concentrations are responsible for the upregulation of *Fxr* gene expression, as a small, but not significant, increase in *Fxr* gene expression was observed following incubation with 30 µM CPZ in the presence of the humanized BA mixture. However, the absence of a larger change in *Fxr* expression might be explained by the fact that different intracellular BAs present at certain concentration ratios can counteract each other’s effect on *Fxr* gene expression. Indeed, some BAs serve as Fxr agonists and some as antagonists (Yu et al. 2002; Paumgartner and Beuers 2004; Dai et al. 2011). In previous studies with rat and human PCLS (Vatakuti et al. 2017; Starokozhko et al. 2017a), it was shown that the *Fxr* pathway was inhibited following 48 h of incubation with cholestatic drugs. However, a significant decrease in *Fxr* expression was not observed in our model. The different composition and relative concentrations of intracellular BAs of mouse PCLS, compared to rat or human PCLS, could be an explanation for the lack of observed *Fxr* gene expression. For example, it was shown that increased levels of GDCA decrease the expression of *Fxr* in human cholangiocarcinoma cells, in vitro (Dai et al. 2011). However, whereas in this study GDCA was not added, and not likely to be synthesized as glycine conjugation is only 0.1% in mouse liver (Alnouti et al. 2008), GDCA was highly increased in rat PCLS (Starokozhko et al. 2017a) upon cholestatic drug exposure. Again, interspecies variation in BA composition may explain at least in part the absence of change in *Fxr* expression in mouse PCLS following incubation with cholestatic compounds.

Interestingly, incubation with the highest concentration of CPZ (30 µM) and humanized BAs increased the gene expression of the BA uptake transporter, *Ntcp*. This was unexpected since *Ntcp* expression is actually expected to decrease when intracellular BA concentrations rise (Kosters and Karpen 2008; Matsubara et al. 2013). The upregulation of *Ntcp* expression was significant in the presence of BAs and a small increase was also observed in the absence of BAs. This upregulation may have led to an increase in the protein expression of Ntcp, thereby facilitating the accumulation of BAs. Upregulation of *Ntcp* gene expression after CPZ treatment is not observed previously.

Finally, we looked at the early fibrosis markers *Col1a1* and *Hsp47*, because cholestasis is thought to progress into liver fibrosis when left untreated (Hirschfield et al. 2010; Yang et al. 2013). As shown in Fig. 5, when PCLS were incubated with the highest concentration of CPZ (30 µM) with the humanized BAs, the fibrosis markers *Col1a1* and *Hsp47* were significantly upregulated. This upregulation indicates that the stellate cells were activated due to hepatoxicity caused by CPZ. *Col1a1* was also upregulated in the absence of BAs, although less pronounced, suggesting that the early onset of fibrogenesis is more pronounced by the presence of BAs. Overall, these results demonstrate that the toxicity related to cholestasis may induce the early onset of fibrosis (Westra et al. 2014). To conclude, downregulation of *Bsep* and upregulation of *Mrp4* gene expression correspond to literature data (Wagner et al. 2003; Szalowska et al. 2013) and could serve as useful markers for the prediction of DIC.

#### Protein expression

Besides looking at gene expression, we measured the changes in protein expression of the export transporter, Bsep, and uptake transporter, Ntcp, following incubation with a non-toxic concentration of a cholestatic drug, or the combination of a cholestatic drug and BAs (Figs. 6, 7). Using the western blot technique, we found that incubation of PCLS with CPZ, significantly without the humanized BA mixture, resulted in reduced Bsep protein expression (Fig. 6a). This decrease was as well observed in the presence of the humanized BA mixture, however not significantly, possibly because the BA mixture itself caused a small reduction in the Bsep protein. Western blot only reveals the amounts of transporters present, not the location or the activity of the transporters. Hence, the results shown in Fig. 6 do not account for competitive inhibition of Bsep by CSA and GB (Noe et al. 2001; Horikawa et al. 2003) or internalization of the Bsep transporter (by CPZ) (Anthérieu et al. 2013). Therefore, the decreased protein expression of Bsep due to incubation with CPZ is suggestive of another mechanism by which CPZ reduces the amount of Bsep protein. Some potential explanations include repressed *Bsep* gene expression or impairment of the renewal of the Bsep protein. Indeed, the gene expression data showed a reduction in *Bsep* expression for all concentrations of CPZ, including the non-toxic concentration (15 μM) used for the western blot experiment. The reduction in gene expression for this CPZ concentration was however not significant (Fig. 5). Therefore, the decrease in Bsep protein expression observed in PCLS, after incubation with CPZ, could also be due to another mechanism than a decrease in *Bsep* gene expression. Interestingly, CSA treatment led to a significant increase in the Bsep protein which was not observed in the presence of the humanized BA mixture. This increase in Bsep protein with CSA treatment is to our knowledge not reported in the literature before. Since no upregulation was seen with qRT-PCR, this increase in Bsep protein expression is likely due to post-translational processes. In the presence of the humanized BA mixture and CSA, a rather decreasing trend was observed for the Bsep protein. Differences between with and without BA treatment could be because of the chemical properties of CSA, whereas BAs ensure proper solubilization of CSA needed for absorption (Stojančević et al. 2013; Pharmacists Association 2021).

Contrary to Bsep, Ntcp protein expression was unaffected by incubation with non-toxic concentrations of all three drugs. This was expected, as Ntcp is thought to decrease as a response to rising intracellular BA concentration (Claudel et al. 2005; Zollner et al. 2006; Halilbasic et al. 2013), and accumulation of BAs with these concentrations was not observed (Fig. 8a–c). The intracellular BA concentrations do increase as the CPZ concentration is increased (Fig. 8a). Additionally, an increase in *Ntcp* gene expression was observed at the highest concentration of CPZ (Fig. 5). Therefore, Ntcp protein expression might be affected at higher concentrations of CPZ than used to obtain the western blot data in Fig. 7.

Concentrations of intracellular BAs.

Lastly, we looked at the accumulation of intracellular BAs (Figs. 8, 9, Table 4), which is thought to cause some of the toxicity observed in DIC (Zollner et al. 2006; Yang et al. 2013). Incubation of PCLS with different concentrations of the cholestatic drug CPZ led to an intracellular accumulation of all measured BAs (Fig. 8a). This effect was also observed without the addition of the humanized BA mixture to the medium, although the concentrations involved are much lower (Fig. 9a). The observed BA accumulation corresponds nicely to the clear reduction of Bsep protein expression observed upon PCLS incubation with CPZ (Fig. 6a). Since Bsep is a primary BA efflux transporter, its reduced expression likely correlates with the intracellular accumulation of BAs. Moreover, the observed accumulation of BAs is also in line with the ATP data, as enhanced toxicity is only observed by incubation with both CPZ and the humanized BA mixture (Fig. 4a). Indeed, the enhanced toxicity observed is likely a result of high concentrations of toxic BAs (Heuman 1989; Delzenne et al. 1992; Bernstein et al. 1999; Song et al. 2011). Furthermore, the accumulation of BAs correlates to the gene expression data, as BA uptake transporter *Ntcp* was upregulated, and BA export transporter *Bsep* was downregulated (Fig. 5). Only PCLS incubated with both CPZ and the humanized BA mixture showed significant upregulation of the early fibrosis markers *Col1a1* and *Hsp47.* This upregulation is likely also caused by BA accumulation accompanied by increased toxicity in hepatocytes leading to the activation of stellate cells. Another cause for the accumulation of BAs could be impaired intracellular communication and leaking of tight junctions (Mottino et al. 2007; Yang et al. 2019). Leaking tight junctions could cause a backflow of BAs from the canaliculi to the hepatocytes, because of the concentration gradient (Yang et al. 2019). However, accumulation due to leaky tight junctions is unlikely in this model, because PCLS have an open biliary compartment towards the culture media, thus there is probably no concentration gradient as is seen in vivo.

In contrast to CPZ, PCLS incubation with CSA and GB either reduced or did not affect the total BA concentration compared to the control. This result deviates from what was observed in rat PCLS (Starokozhko et al. 2017a), where all three drugs led to an accumulation of intracellular BAs. The main difference in BA composition between mouse and rat PCLS, after incubation with CSA and GB, is the intracellular concentration of GCA. This difference may be explained by differences in the endogenous BA pools of mice and rats. Indeed, the GCA concentration in rat tissue is 42-times higher than in mice (14.0 pmol/mg compared to 0.33 pmol/mg) (García-Cañaveras et al. 2012). Therefore, our BA mixture contained 0.03 µM GCA while the BA mixture used by Starokozhko et al. (2017a) contained 0.63 µM GCA. Based on pilot toxicity experiments performed with BA mixtures in mouse PCLS, the concentration of our total BA mixture was 16 µM, while the BA mixture used by Starokozhko et al. (2017a) was 60 µM. Although we added fewer BAs to the incubation medium, the concentration of most of the intracellular BAs measured was still higher in mouse PCLS than what was observed in rat PCLS. The GCA concentration, however, was significantly lower, with 20.3 pmol/mg protein in our mouse PCLS compared to 156.5 pmol/mg protein in rat PCLS (Starokozhko et al. 2017a). Moreover, incubation of rat PCLS with GB and CSA resulted in an increase of the intracellular GCA concentration from 156.5 to 364.3 and to 1275.6 pmol/mg protein, respectively (Starokozhko et al. 2017a). Incubation of mouse PCLS with CSA and GB changed the GCA concentration from 20.3 to 5.7 and to 22.4 pmol/mg protein, respectively. The absence of GCA accumulation in mouse PCLS may be explained by the fact that mice predominantly conjugate BAs with taurine (Alnouti et al. 2008). The cholestatic toxicity observed in rat PCLS (Starokozhko et al. 2017a) by CSA and GB could therefore be due to the accumulation of intracellular BAs, mainly GCA, which we did not observe in our mouse model. Human liver tissue however contains much less GCA, namely 2.7 pmol/mg protein (García-Cañaveras et al. 2012). Therefore, the mouse model with 20.3 pmol/mg GCA better reflects the human in vivo situation than the rat model with 156.5 pmol/mg GCA. Still, the lack of other glycine-conjugated BAs makes this mouse model less suitable to study human DIC. To improve this mouse model, the concentration of GCA can be reduced, and other glycine-conjugated BAs like GDCA, and GCDCA should be added.

Another explanation for the absence of BA accumulation after CSA and GB incubation may be the different mechanisms of these drugs in the inhibition of bile transport in mice because of interspecies variation in the Bsep protein function (Green et al. 2000; Horikawa et al. 2003). Since CSA and GB inhibit Bsep directly (Noe et al. 2001; Horikawa et al. 2003), interspecies variation could lead to reduced drug-protein affinity and therefore ineffective Bsep inhibition. In contrast, CPZ inhibits Bsep indirectly (Anthérieu et al. 2013; Zhang et al. 2016) and incubation with this compound resulted in BA accumulation in this mouse model. Although the amino acid sequence of mouse Bsep is highly similar to the human and rat Bsep, and shares 89% to the human Bsep and 94% to the rat Bsep protein, significant differences are present in the Bsep protein between mouse, rat and human (Green et al. 2000). Still, CSA and GB are found to competitively inhibit the mouse Bsep protein (Noe et al. 2001). Interspecies differences are therefore unlikely, but this needs to be tested with human PCLS.

Even though the total concentration of BAs is not increased by incubation with CSA and GB, the intracellular concentrations of DCA and its conjugates (HDCA, TDCA) are increased by all drugs. CPZ, CSA and GB increased DCA from 52 to 297, 107, and 112 pmol/mg protein, respectively. Starokozhko et al. (2017a) also found an increase in intracellular DCA and its conjugates following separate incubation of rat PCLS with all three drugs. Since DCA is known to accumulate in cholestasis, causing severe toxicity (Delzenne et al. 1992; Bernstein et al. 1999), the DCA increase we observed could be used as an early marker for cholestasis development (Yang et al. 2019). It is likely that enhanced toxicity in PCLS incubated with CSA or GB combined with the humanized BA mixture is not manifested because DCA and other BAs did not accumulate to toxic levels. In contrast, CPZ treatment increased all BA concentrations and resulted in the largest DCA increase of the three drugs. This suggests that a higher amount of DCA and/or accumulation of the other BAs is necessary to induce cholestasis-related toxicity. Increasing incubation times and/or concentrations of CSA and GB might be necessary to observe enhanced toxicity by the addition of the humanized BA mixture.

## Conclusion

In this study, we have generated a complex model based on mouse liver slices to study the onset and progression of DIC. The organotypic nature of our model means that it is possible to consider organ response to drugs known to induce cholestasis in humans. Our model thus represents the multifactorial nature of cholestasis. An added benefit is the possibility to humanize this rodent model through incubation with a physiologically representative mixture of BAs found in both mice and humans, thus providing a means to use a more generally available mouse model to study DIC development as it occurs in humans. This model should in the future serve as a substitute for mouse in vivo models, like the knock-out Cyp2c70 mouse model (Boer et al. 2019), reducing and refining animal testing and resulting in less discomfort for animals. Moreover, this model can be used to investigate DIC even further by modifying the mouse DNA, with the use of knock-out mice, or other gene silencing techniques, like small interfering RNA (Ruigrok et al. 2018).

We conclude that the onset and the development of DIC can be investigated in vitro by considering four indicators, namely 1) a reduction in the number of a given BA efflux transporter (especially Bsep) or repression of the corresponding gene (Abcb11) by cholestatic drugs, and 2) accumulation of intracellular BAs (mainly DCA and its conjugates), followed by 3) adaptive responses in the gene expression of certain BA efflux transporters (e.g. *Mrp4* upregulation) and 4) by measuring cholestatic injury through the activation of other cell types (e.g. *Hsp47* and *Col1a1* upregulation specifically in hepatic stellate cells). In this study, CPZ appears to be the most effective drug to rapidly induce cholestasis in mouse PCLS and can be used in future studies to advance the cholestatic model. Indications are that the approach used to induce cholestasis in mouse liver slices could be further developed to study the onset and progression into fibrosis. Eventually, this cholestatic model can be applied to human PCLS, eliminating interspecies variation and allowing detection of DIC in the early stages of drug development. Moreover, this cholestatic model can serve as a benchmark for future research with mouse PCLS, for example in the development of advanced imaging methods to investigate the mechanisms underlying DIC in real-time with techniques like nuclear magnetic resonance and imaging mass spectrometry.

## Supplementary Information

Below is the link to the electronic supplementary material.

Supplementary file1 (PDF 770 KB)
