# Supplementary material for: Mouse precision-cut liver slices as an ex vivo model to study drug-induced cholestasis
Source: Arch Toxicol. 2022 Jun 16;96(9):2523–43. doi: 10.1007/s00204-022-03321-2 (PMC9325861; doi:10.1007/s00204-022-03321-2)
Supplement: Supplementary file 1 — Supplementary file1 (PDF 770 KB) [file 204_2022_3321_MOESM1_ESM.pdf]

SUPPLEMENTARY INFORMATION

Mouse PCLS as an *ex vivo* model to study drug-induced cholestasis

R.E.H. Karsten[1], N.J.W. Krijnen[1], W. Maho[2], H.P. Permentier[2], E. Verpoorte[1], P. Olinga. [3]

[1] University of Groningen, Groningen Research Institute of Pharmacy, Department of Pharmaceutical Analysis, Antonius Deusinglaan 1, 9713 AV Groningen, the Netherlands

[2] University of Groningen, Groningen Research Institute of Pharmacy, Department of Analytical Biochemistry and Interfaculty Mass Spectrometry Center, A. Deusinglaan 16, 9713 AV Groningen, The Netherlands

[3] University of Groningen, Groningen Research Institute of Pharmacy, Department of Pharmaceutical Technology and Biopharmacy, Antonius Deusinglaan 1, 9713 AV Groningen, the Netherlands

Table of Contents

Table S1 Primer sequences used for relative gene expression analysis by qRT-PCR S-2

Table S2 Intracellular concentrations of 11 different bile acids (BAs) in PCLS incubated for 0h or 48h in the presence or absence of the humanized BA mixture. S-2

Table S3 Nonlinear regression analysis of normalized concentration-response curves S-3

Table S4 Intracellular concentrations of 11 different bile acids (BAs) in PCLS incubated for 48h in the presence of the humanized BA mixture, and with or without a cholestatic drug S-4

Table S5 Intracellular concentrations of 11 different bile acids (BAs) in PCLS incubated for 48h in the absence of the humanized BA mixture, and with or without a cholestatic drug S-5

Fig. S1 Western blot bands of chlorpromazine (CPZ) treated PCLS. S-6

Fig. S2 Western blot bands of cyclosporin (CSA) treated PCLS. S-7

Fig. S3 Western blot bands of glibenclamide (GB) treated PCLS. S-8

Table S1 *Primer sequences used for relative gene expression analysis by qRT-PCR*

**Target protein (gene code)**	**Primer**	**Sequence (5' to 3')**
Bsep **(Abcb11)**	Forward	ACACCATTGTATGGATCAACAGC
	Reverse	CACCAACTCCTGCGTAGATGC
Mrp2 **(Abcc2)**	Forward	AGCAGGTGTTCGTTGTGTGT
	Reverse	CAGGAGGAATTGTGGCTTGTC
Mrp3 **(Abcc3)**	Forward	CTGGGTCCCCTGCATCTAC
	Reverse	GCCGTCTTGAGCCTGGATAAC
Mrp4 **(Abcc4)**	Forward	GGCACTCCGGTTAAGTAACTC
	Reverse	TGTCACTTGGTCGAATTTGTTCA
Ntcp **(Slc10a1)**	Forward	ATGGAGGCGCACAACGTATC
	Reverse	ACTACCAGAATGACGCTGAGC
Fxr **(Nr1h4)**	Forward	GCTTGATGTGCTACAAAAGCTG
	Reverse	CGTGGTGATGGTTGAATGTCC
Cyp7a1 **(Cyp7a1)**	Forward	GGGATTGCTGTGGTAGTGAGC
	Reverse	GGTATGGAATCAACCCGTTGTC
Col1a1 **(Col1a1)**	Forward	TGACTGGAAGAGCGGAGAGT
	Revers	ATCCATCGGTCATGCTCTCT
Hsp47 **(Serpinh1)**	Forward	AGGTCACCAAGGATGTGGA
	Reverse	CAGCTTCTCCTTCTCGTCGT
**Reference gene**	Forward	TTACTTGGCCGAGGTTGCT
**(Ywhaz)**	Reverse	TGCTGTGACTGGTCCACAAT

Table S2 Intracellular concentrations of 11 different bile acids (BAs) in PCLS incubated for 0h or 48h in the presence or absence of the humanized BA mixture. Data are expressed as mean absolute values (pmol BA per mg protein) of 5 independent experiments (n=5 mice) using three slices per condition ± SEM. The percent of each BA in the total amount of measured BA is given in parentheses

**pmol/mg protein**	**0h**	**48h**	**48h + BA**
**TCA**	213.0±42.8 (79.1%)	0.6±0.1 (5.5%)	177.7±54.8 (33.6%)
**TCDCA**	15.8±4.1 (5.9%)	7.3±1.0 (67.1%)	7.2±1.2 (1.4%)
**TDCA**	14.8±1.1 (5.5%)	0.0±0.0 (0.2%)	8.2±1.3 (1.6%)
**THDCA**	9.5±1.3 (3.5%)	1.9±0.3 (17.2%)	5.5±0.4 (1.0%)
**CA**	10.4±2.4 (3.9%)	0.4±0.1 (3.7%)	206.2±17.6 (39.0%)
**CDCA**	1.1±0.3 (0.4%)	0.2±0.1 (2.0%)	16.3±1.8 (3.1%)
**DCA**	1.0±0.5 (0.4%)	0.1±0.1 (0.8%)	52.0±7.0 (9.8%)
**HDCA**	0.5±0.3 (0.2%)	0.0±0.0 (0.0%)	2.5±1.7 (0.5%)
**LCA**	2.4±0.6 (0.9%)	0.1±0.1 (1.3%)	1.5±0.3 (0.3%)
**UDCA**	0.3±0.1 (0.1%)	0.2±0.1 (2.3%)	31.9±3.2 (6.0%)
**GCA**	0.3±0.1 (0.1%)	0.0±0.0 (0.0%)	20.3±6.8 (3.8%)

**Total**	269.3±0.0 (100%)	10.9±0.0 (100%)	529.3±82.3 (100%)

Table S3 *Nonlinear regression analysis of normalized concentration-response curves used to find the best-fit curve for PCLS incubated with and without drugs, in the presence (with) or absence (w/o) of the humanized bile acid (BA) mixture (Figure 4). The best-fit curve is used to calculate the inhibitory concentrations (IC) of drug that result in 5%, 25% and 50% decrease in PCLS ATP content compared to control (PCLS incubated without drug or BA). Best-fit values of IC5, IC25, and IC50 concentrations for chlorpromazine (CPZ) are different with and without BA. Best-fit values for IC5 and IC25 for cyclosporin A (CSA) are different in the presence or absence of BA, while the IC50 is the same for CSA with and without BA. Best-fit values for the IC5, IC25 and IC50 concentrations of glibenclamide (GB) are the same with or without BA. HS is the hillslope of the corresponding concentration-response curve*

	CPZ			***CSA***				***GB***		
IC	*IC different with and w/o BA*		*IC the same with and w/o BA*	*IC different with and w/o BA*		*IC the same with and w/o BA*		*IC different with and w/o BA*		*IC the same with and w/o BA*
	w/o BA	*with BA*		*w/o BA*	*with BA*	*w/o BA*	*with BA*	*w/o BA*	*with BA*	
50%	*29.1*	*21.55*	*-*	*-*	*-*	*9.5*	*9.5*	*-*	*-*	*69.7*
25%	*22.9*	*17.0*	*-*	*1.8*	*6.0*	*-*	*-*	*-*	*-*	*55.5*
5%	*15.4*	*11.4*	*-*	*0.2*	*2.5*	*-*	*-*	*-*	*-*	*37.9*
HS	*-4.6*	*-4.6*	*-*	*-0.8*	*-2.1*	*-0.7*	*-2.1*	*-*	*-*	*-4.8*

Table S4 *Intracellular concentrations of 11 different bile acids (BAs) in PCLS incubated for 48h in the presence of the humanized BA mixture, and with or without a cholestatic drug: chlorpromazine (CPZ) (15, 30 µM), cyclosporin A (CSA) (1, 6 µM), or glibenclamide (GB) (25, 65 µM). Data for individual BA intracellular concentrations are expressed as mean absolute values (pmol BA per mg protein) ± SEM for 5 independent experiments (n=5 mice), using three slices per mouse for each condition. The percent of each BA in the total amount of measured BA is given in parentheses*

**pmol/mg protein**	**BA control**	**CPZ 15µM**	**CPZ 30µM**	**CSA 1µM**	**CSA 6µM**	**GB 25µM**	**GB 65µM**
**TCA**	177.7±54.8 (33.6%)	244.1±42.8 (33.8%)	351.2±67.0 (29.8%)	96.0±19.3 (29.4%)	33.3±2.0 (10.3%)	103.8±8.6 (30.6%)	135.2±19.9 (26.4%)
**TCDCA**	7.2±1.2 (1.4%)	10.1±0.2 (1.4%)	18.2±5.3 (1.5%)	6.7±0.5 (2.0%)	3.6±0.4 (1.1%)	7.7±0.5 (2.3%)	8.6±1.2 (1.7%)
**TDCA**	8.2±1.3 (1.6%)	14.1±0.8 (2.0%)	44.3±5.1 (3.8%)	9.2±1.2 (2.8%)	18.8±2.3 (5.9%)	7.6±0.8 (2.2%)	14.6±0.9 (2.9%)
**THDCA**	5.5±0.4 (1.0%)	9.4±1.4 (1.3%)	9.0±3.0 (0.8%)	5.2±0.4 (1.6%)	4.4±1.0 (1.4%)	8.3±1.4 (2.4%)	11.4±2.6 (2.2%)
**CA**	206.2±17.6 (39.0%)	259.9±32.0 (36.0%)	314.1±29.7 (26.7%)	92.8±10.9 (28.5%)	78.8±4.9 (24.5%)	103.6±10.3 (30.5%)	132.3±11.3 (25.9%)
**CDCA**	16.3±1.8 (3.1%)	20.9±1.8 (2.9%)	41.3±6.4 (3.55%)	14.1±2.1 (4.3%)	19.3±2.6 (6.05%)	14.7±1.6 (4.3%)	14.8±1.3 (2.9%)
**DCA**	52.0±7.0 (9.8%)	87.7±14.0 (12.1%)	297.9±25.1 (25.3%)	50.6±10.9 (15.5%)	106.9±18.8 (33.2%)	50.7±7.4 (14.9%)	121.3±8.6 (23.7%)
**HDCA**	2.5±1.7 (0.5%)	4.0±2.5 (0.6%)	13.6±3.9 (1.2%)	3.3±2.0 (1.0%)	12.9±1.8 (4.0%)	9.4±2.7 (2.8%)	25.7±1.5 (5.0%)
**LCA**	1.5±0.3 (0.3%)	3.4±1.3 (0.5%)	14.9±8.6 (1.3%)	2.6±0.9 (0.8%)	8.9±4.3 (2.8%)	0.8±0.5 (0.2%)	0.9±0.3 (0.2%)
**UDCA**	31.9±3.2 (6.0%)	34.2±4.0 (4.7%)	46.3±3.0 (3.9%)	27.9±1.9 (8.5%)	28.9±1.6 (9.0%)	19.1±1.7 (5.6%)	24.5±3.9 (4.8%)
**GCA**	20.3±6.8 (3.8%)	34.7±16.0 (4.8%)	26.1±3.5 (2.2%)	17.9±6.1 (5.5%)	5.7±1.0 (1.8%)	13.4±2.4 (4.0%)	22.4±6.1 (4.4%)
**Total**	529.3±82.3 (100%)	722.5±95.4 (100%)	1177.0±113.2 (100%)	326.1±50.8 (100.0%)	321.7±29.7 (100%)	339.2±20.7 (100%)	511.7±43.6 (100%)

Table S5 *Intracellular concentrations of 11 different bile acids (BAs) in PCLS incubated for 48h in the absence of the humanized BA mixture, and with or without a cholestatic drug; chlorpromazine (CPZ) (15, 30 µM), cyclosporin A (CSA) (1, 6 µM), or glibenclamide (GB) (25, 65 µM). Data for individual BA intracellular concentrations are expressed as mean absolute values (pmol/mg protein) ± SEM for 5 independent experiments (n=5 mice), using three slices per mouse for each condition. The percent of each BA in the total amount of measured BA is given in parentheses*

pmol/mg protein	Control	CPZ 15µM	CPZ 30µM	CSA 1µM	CSA 6µM	GB 25µM	GB 65µM
**TCA**	0.6±0.1 (5.5%)	0.8±0.1 (4.5%)	1.2±0.1 (3.6%)	0.4±0.1 (7.0%)	0.1±0.1 (4.7%)	0.7±0.2 (10.5%)	0.3±0.1 (6.3%)
**TCDCA**	7.3±1.0 (67.1%)	14.4±1.7 (77.4%)	25.0±2.2 (76.5%)	3.8±0.8 (64.9%)	1.1±0.2 (42.3%)	2.3±0.5 (36.6%)	2.0±0.4 (48.5%)
**TDCA**	0.0±0.0 (0.2%)	0.1±0.1 (0.7%)	0.1±0.1 (0.2%)	0.0±0.0 (0.4%)	0.1±0.1 (4.7%)	0.0±0.0 (0.4%)	0.0±0.0 (0.2%)
**THDCA**	1.9±0.3 (17.2%)	2.4±0.3 (12.8%)	5.2±0.7 (16.0%)	0.7±0.1 (12.1%)	0.1±0.0 (4.5%)	1.8±0.3 (28.8%)	0.5±0.1 (11.8%)
**CA**	0.4±0.1 (3.7%)	0.3±0.0 (1.8%)	0.4±0.1 (1.1%)	0.4±0.1 (7.0%)	0.6±0.3 (24.3%)	0.5±0.0 (8.2%)	0.5±0.2 (13.1%)
**CDCA**	0.2±0.1 (2.0%)	0.2±0.2 (1.1%)	0.4±0.2 (1.1%)	0.3±0.1 (4.4%)	0.2±0.1 (6.0%)	0.2±0.1 (3.7%)	0.4±0.3 (10.1%)
**DCA**	0.1±0.1 (0.8%)	0.1±0.1 (0.4%)	0.1±0.1 (0.4%)	0.1±0.1 (1.4%)	0.1±0.0 (2.0%)	0.6±0.5 (9.4%)	0.1±0.1 (3.4%)
**HDCA**	0.0±0.0 (0.0%)	0.0±0.0 (0.0%)	0.0±0.0 (0.0%)	0.0±0.0 (0.0%)	0.0±0.0 (0.5%)	0.0±0.0 (0.0%)	0.0±0.0 (0.9%)
**LCA**	0.1±0.1 (1.3%)	0.2±0.1 (1.2%)	0.3±0.2 (1.1%)	0.2±0.1 (2.9%)	0.3±0.2 (10.7%)	0.1±0.1 (2.4%)	0.2±0.1 (5.5%)
**UDCA**	0.2±0.1 (2.3%)	0.0±0.0 (0.0%)	0.0±0.0 (0.0%)	0.0±0.0 (0.0%)	0.0±0.0 (0.2%)	0.0±0.0 (0.0%)	0.0±0.0 (0.0%)
**GCA**	0.0±0.0 (0.0%)	0.0±0.0 (0.0%)	0.0±0.0 (0.0%)	0.0±0.0 (0.0%)	0.0±0.0 (0.0%)	0.0±0.0 (0.0%)	0.0±0.0 (0.0%)
**Total**	10.9±0.0 (100%)	18.7±0.0 (100%)	32.7±0.0 (100%)	5.8±0.0 (100%)	2.7±0.0 (100%)	6.3±0.0 (100%)	4.2±0.0 (100%)

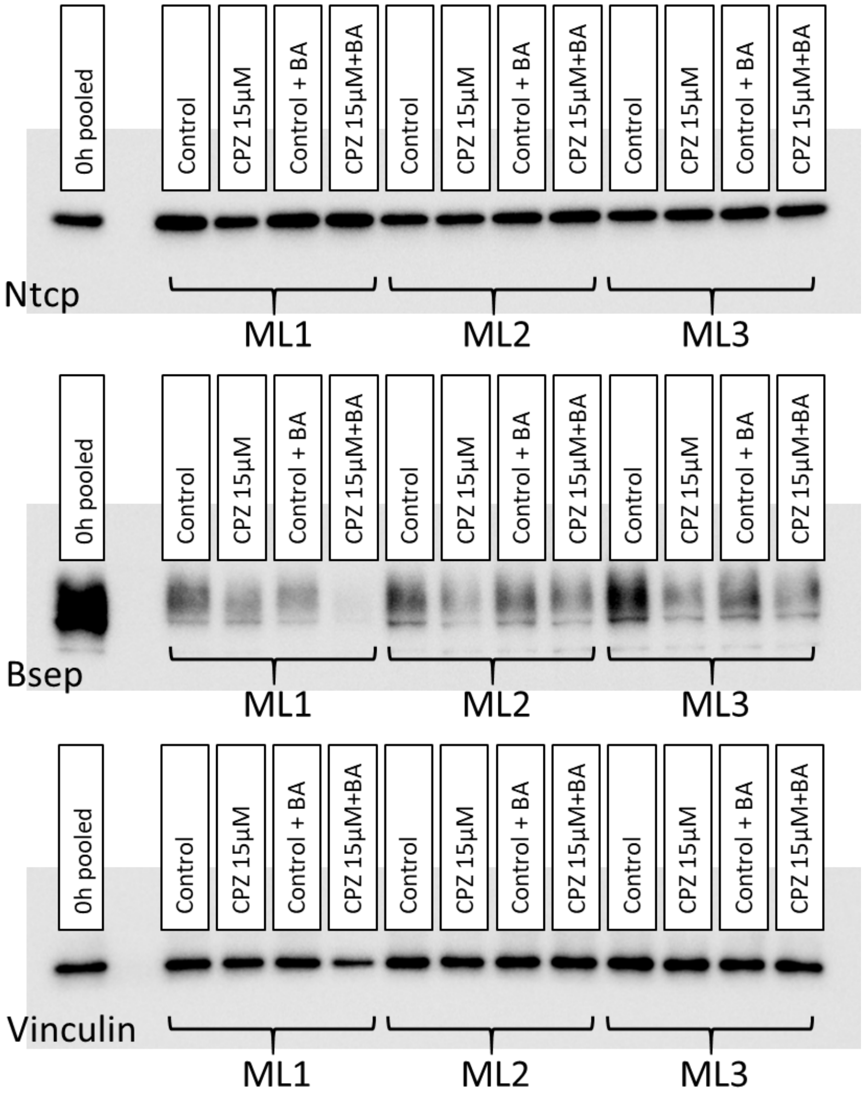

Fig. S1 *Western blot bands of chlorpromazine (CPZ) treated PCLS. Shown is the protein expression of sodium-taurocholate cotransporting polypeptide (Ntcp), bile salt export pump (Bsep), and the loading control, vinculin, of mouse PCLS incubated for 48h with a non-toxic concentration of CPZ (15 µM) in the presence or absence of humanized bile acid (BA) mixture. Western blot bands are shown for measurements made with three individual mice (n=3 mice; ML1, ML2 and ML3) using three slices per mouse pooled for each condition*

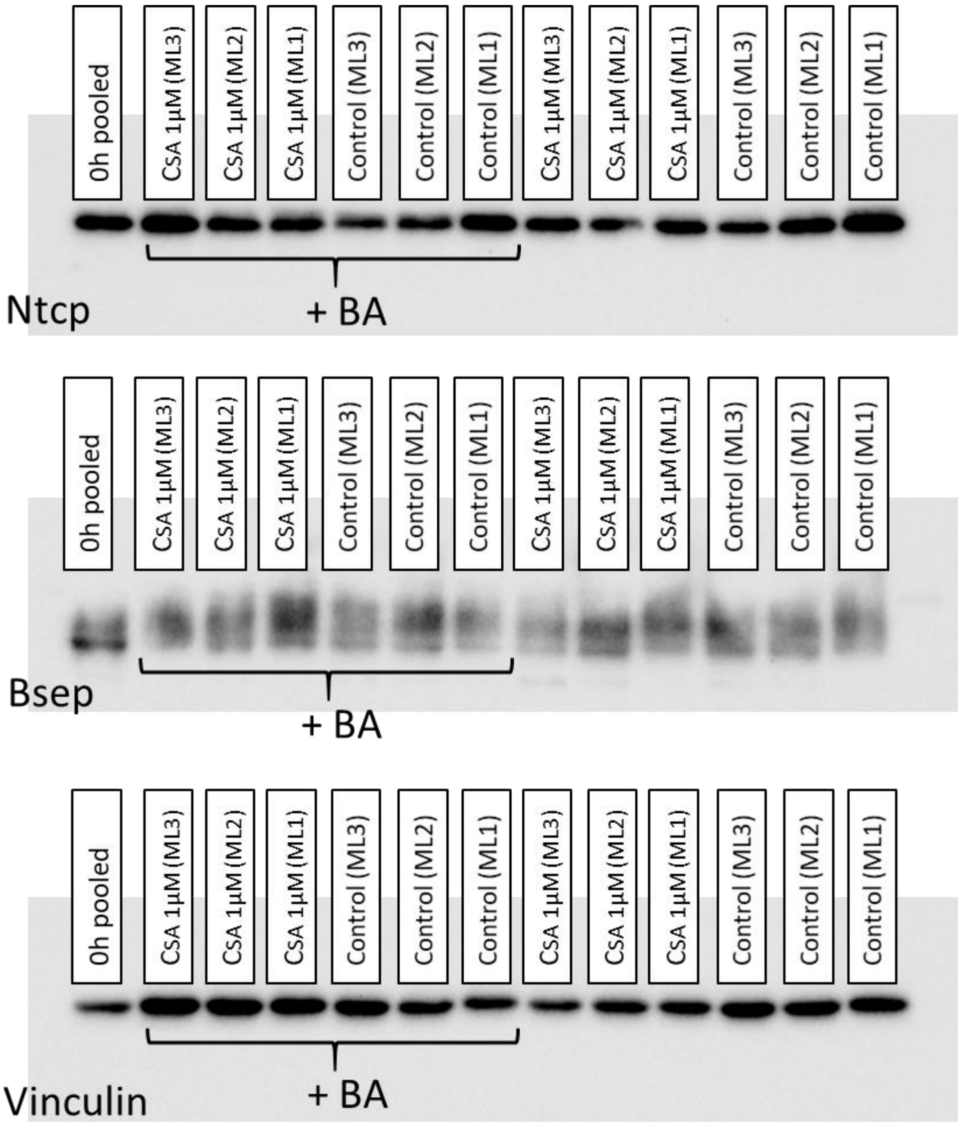

Fig. S2 *Western blot bands of cyclosporin (CSA) treated PCLS. Shown is the protein expression of sodium-taurocholate cotransporting polypeptide (Ntcp), bile salt export pump (Bsep), and the loading control, Vinculin, for mouse PCLS incubated for 48h with a non-toxic concentration of CSA (1 µM) in the presence or absence of the humanized bile acid (BA) mixture. Western blot bands are shown for measurements made with three individual mice (n=3 mice; ML1, ML2 and ML3) using three slices pooled for each condition*

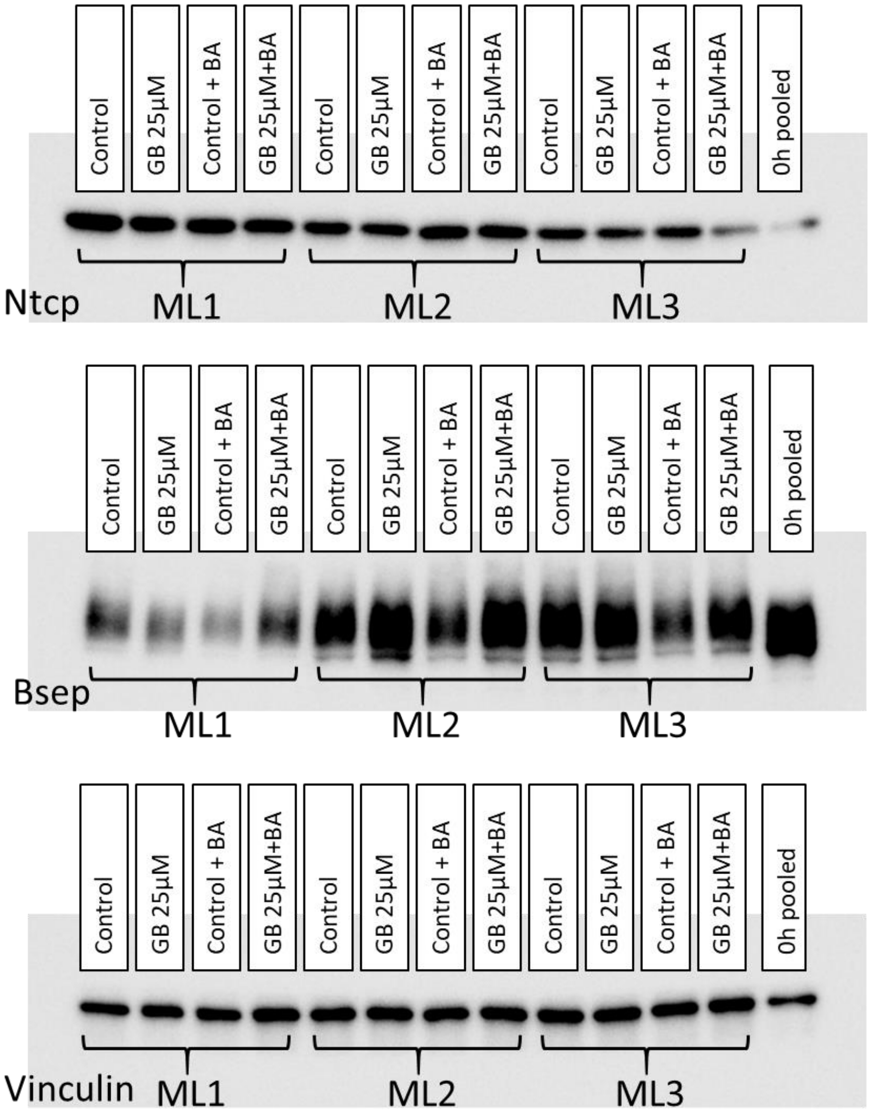

Fig. S3 *Western blot bands of glibenclamide (GB) treated PCLS. Shown is the protein expression of sodium-taurocholate cotransporting polypeptide (Ntcp), bile salt export pump (Bsep), and the loading control, Vinculin, for mouse PCLS incubated for 48h with a non-toxic concentration of glibenclamide (25 µM)(GB) in the presence or absence of the humanized bile acid (BA) mixture. Western blot bands are shown for measurements made with three individual mice (n=3 mice; ML1, ML2 and ML3) using three slices per mouse pooled for each condition*
